# Colon and rectal cancer treatment patterns and their associations with clinical, sociodemographic and lifestyle characteristics: analysis of the Australian 45 and Up Study cohort

**DOI:** 10.1186/s12885-023-10528-8

**Published:** 2023-01-18

**Authors:** Sarsha Yap, Emily He, Sam Egger, David E Goldsbury, Jie-Bin Lew, Preston J Ngo, Joachim Worthington, Hannah Rillstone, John R Zalcberg, Jeff Cuff, Robyn L Ward, Karen Canfell, Eleonora Feletto, Julia Steinberg

**Affiliations:** 1 The Daffodil Centre, The University of Sydney, a joint venture with Cancer Council NSW, 153 Dowling St, Woolloomooloo, Sydney, New South Wales 2011 Australia; 2 Cancer Policy and Advocacy, Cancer Council NSW, Woolloomooloo, Sydney, New South Wales Australia; 3 School of Public Health and Preventive Medicine, Monash University, Melbourne, Victoria Australia; 4 Department of Medical Oncology, Alfred Health, Melbourne, Victoria Australia; 5 Faculty of Science Biotech and Biomolecular Science, University of New South Wales, Sydney, New South Wales Australia; 6 Research Advocate, The Daffodil Centre, Sydney, New South Wales Australia; 7 Faculty of Medicine and Health, University of Sydney, Sydney, New South Wales Australia

**Keywords:** Colon cancer, Rectal cancer, Cancer treatment, Radiotherapy, Chemotherapy, Cancer surgery, Australia, 45 and up Study cohort

## Abstract

**Background:**

Colorectal cancer is the third most diagnosed cancer globally and the second leading cause of cancer death. We examined colon and rectal cancer treatment patterns in Australia.

**Methods:**

From cancer registry records, we identified 1,236 and 542 people with incident colon and rectal cancer, respectively, diagnosed during 2006-2013 in the 45 and Up Study cohort (267,357 participants). Cancer treatment and deaths were determined via linkage to routinely collected data, including hospital and medical services records. For colon cancer, we examined treatment categories of “surgery only”, “surgery plus chemotherapy”, “other treatment” (i.e. other combinations of surgery/chemotherapy/radiotherapy), “no record of cancer-related treatment, died”; and, for rectal cancer, “surgery only”, “surgery plus chemotherapy and/or radiotherapy”, “other treatment”, and “no record of cancer-related treatment, died”. We analysed survival, time to first treatment, and characteristics associated with treatment receipt using competing risks regression.

**Results:**

86.4% and 86.5% of people with colon and rectal cancer, respectively, had a record of receiving any treatment ≤2 years post-diagnosis. Of those treated, 93.2% and 90.8% started treatment ≤2 months post-diagnosis, respectively. Characteristics significantly associated with treatment receipt were similar for colon and rectal cancer, with strongest associations for spread of disease and age at diagnosis (*p*<0.003). For colon cancer, the rate of “no record of cancer-related treatment, died” was higher for people with distant spread of disease (versus localised, subdistribution hazard ratio (SHR)=13.6, 95% confidence interval (CI):5.5-33.9), age ≥75 years (versus age 45-74, SHR=3.6, 95%CI:1.8-7.1), and visiting an emergency department ≤1 month pre-diagnosis (SHR=2.9, 95%CI:1.6-5.2). For rectal cancer, the rate of “surgery plus chemotherapy and/or radiotherapy” was higher for people with regional spread of disease (versus localised, SHR=5.2, 95%CI:3.6-7.7) and lower for people with poorer physical functioning (SHR=0.5, 95%CI:0.3-0.8) or no private health insurance (SHR=0.7, 95%CI:0.5-0.9).

**Conclusion:**

Before the COVID-19 pandemic, most people with colon or rectal cancer received treatment ≤2 months post-diagnosis, however, treatment patterns varied by spread of disease and age. This work can be used to inform future healthcare requirements, to estimate the impact of cancer control interventions to improve prevention and early diagnosis, and serve as a benchmark to assess treatment delays/disruptions during the pandemic. Future work should examine associations with clinical factors (e.g. performance status at diagnosis) and interdependencies between characteristics such as age, comorbidities, and emergency department visits.

**Supplementary Information:**

The online version contains supplementary material available at 10.1186/s12885-023-10528-8.

## Background

Colorectal cancer (CRC) is the third most commonly diagnosed cancer globally, and the second leading cause of cancer death [1, 2]. CRC incidence and mortality rates have been decreasing in high income countries, which has been largely attributed to the early detection of pre-cancerous lesions (polyps) or early-stage cancer through screening and advancements in treatment [3–5]. However, the numbers of cases and deaths remain high due to an ageing population, leading to substantial healthcare requirements and costs. Moreover, in high-income countries, CRC incidence rates are increasing in adults aged <50 years [6].

In Australia, a high-income country with universal healthcare supplemented by private health insurance, cancer accounts for the third highest disease expenditure to the health system [7], and CRC was the costliest cancer type to treat in 2013 [8]. CRC treatment costs are particularly high in the first year after diagnosis compared to other cancer types [8], and healthcare costs and utilisation are higher for those with advanced spread of disease [9]. Understanding CRC treatment patterns and their association with specific characteristics of people with colon and rectal cancer (e.g. age, cancer stage at diagnosis) is important for estimating future healthcare requirements and the current and future impact of cancer control interventions.

To date, Australian data on colon and rectal cancer treatment patterns are limited and focus on specific treatments [10–16]. Moreover, most existing studies have examined colon and rectal cancers combined, while the management and treatment of these cancer types differ [17–19]. There are also limited Australian data on the time from a CRC diagnosis to first cancer treatment. A recent systematic review found that system-level delays in cancer treatment are associated with increased cancer mortality [20]. In view of treatment disruptions that occurred during the COVID-19 pandemic, benchmarks of pre-pandemic time to treatment are important to estimate the impact of treatment delays.

In this study, we examined cancer treatment patterns for people with incident colon cancer and separately, incident rectal cancer in the 45 and Up Study, a large cohort of Australian residents. We describe treatment patterns for specific treatment combinations by spread of disease at diagnosis, estimate time from diagnosis to first treatment received, and identify associations between characteristics of people with colon and rectal cancer and treatment receipt.

## Methods

### Study cohort

We utilised the Sax Institute’s 45 and Up Study cohort of 267,357 participants from New South Wales (NSW), Australia, recruited from 2005 to 2009 [21, 22]. Briefly, adults aged ≥45 years were randomly sampled from the Services Australia Medicare enrolment database (MEDB) if they had received medical care within the previous two years [22]. MEDB has near-complete coverage of the population. People living in remote and rural areas and those aged ≥80 years were oversampled. Overall, the response rate was ~19% and the cohort represents ~11% of the NSW population aged ≥45 years. Participants completed a questionnaire containing health, lifestyle and sociodemographic information at baseline [23], and consented to be followed-up through questionnaires and linkage to routinely collected administrative health databases.

Baseline questionnaire information was linked to health databases to identify incident cancers, deaths, and cancer treatment received (i.e., surgery, chemotherapy and radiotherapy), as well as clinical, sociodemographic and lifestyle characteristics of people with cancer. We linked to available data at the time of linkage, including: (a) NSW Cancer Registry (NSWCR; Jan-1994 to Dec-2013) to ascertain colon and rectal cancers diagnosed after baseline according to the International Classification of Diseases 10 Australian Modification (ICD-10 AM) [24], some characteristics of people with cancer (see below), and cause of death after diagnosis; (b) Registry of Births, Deaths and Marriages (RBDM; Jan-2006 to Jun-2017) to identify deaths after diagnosis; (c) Emergency Department Data Collection (EDDC; Jan-2005 to Dec-2013) to identify emergency department visits pre-diagnosis; (d) Admitted Patient Data Collection (APDC; Jul-2001 to Dec-2015) to identify comorbidities pre-diagnosis and cancer treatment received in hospital post-diagnosis; (e) Medicare Benefits Schedule (Jan-2006 to Dec-2016) to identify other cancer treatment received post-diagnosis and (f) Pharmaceutical Benefits Scheme (Jan-2006 to Dec-2016) to identify government-subsidised chemotherapy received post-diagnosis. Databases (a)-(d) were probabilistically linked by the Centre for Health Record Linkage (CHeReL) [25]. Databases (e)-(f) were supplied by Services Australia and deterministically linked by the Sax Institute using a unique identifier provided by Services Australia.

### Ascertainment of people with cancer

We excluded cohort participants who withdrew, were part of the pilot study, <45 years old, a Department of Veterans' Affairs client (as their available treatment data are incomplete), had a self-reported cancer diagnosed prior to 1994, or with linkage errors. We identified incident colon cancers (ICD-10 AM code C18) and rectal cancers (C19-20) using NSWCR records, and excluded those with multiple cancer diagnoses, a cancer diagnosis before or at baseline, exact month of diagnosis not recorded, or cancer diagnosis based on death certificate only.

### Characteristics of people with cancer

A total of 16 characteristics were examined. We obtained age, spread of disease, place of residence (using the Accessibility and Remoteness Index of Australia plus [ARIA+] [26]) and area-level socioeconomic status (SES; using the Index of Relative Socio-Economic Disadvantage (IRSD) [27]) from NSWCR, all at time of cancer diagnosis. From the self-reported baseline questionnaire, we obtained sex, smoking status, body mass index (kg/m^2^), highest educational qualification, private health insurance status, marital status, language other than English spoken at home, Medical Outcomes Study Physical Functioning 10 (MOSPF-10) score [28, 29], faecal occult blood testing (FOBT) history (ever/never), and sigmoidoscopy/colonoscopy history (ever/never). Charlson’s Comorbidity Index was derived from APDC records using the ICD-10-AM codes specified by [30] and conditions scored as in [31], capturing conditions in the 5 years pre-diagnosis (Additional file 1). Emergency department visits from EDDC records were captured for the 31 days pre-diagnosis.

### Cancer treatment utilisation

We focused on surgery, chemotherapy or radiotherapy treatment in the 0-2 years after diagnosis (codes are listed in Additional file 2). We considered all treatment combinations, or no record of these cancer-related treatments ≤2 years post-diagnosis. Treatment combinations were grouped into five categories for each cancer type to ensure a sufficient sample size (Additional file 3). For colon cancer, categories included “surgery only” and “surgery plus chemotherapy”; and for rectal cancer, “surgery only” and “surgery plus chemotherapy and/or radiotherapy”.

Treatment combinations containing smaller sample sizes were grouped into the “other treatment” category: for colon and rectal cancer, this included i) chemotherapy only, ii) radiotherapy only, iii) chemotherapy plus radiotherapy; for colon cancer, it additionally included iv) surgery plus radiotherapy, v) surgery plus chemotherapy plus radiotherapy. For both cancers, we also considered categories of “no record of cancer-related treatment, died”, and “no record of cancer-related treatment, alive” based on the same period (2 years post-diagnosis), hereafter referred to as “no treatment, died” and “no treatment, alive” categories, respectively.

### Statistical analyses

Analyses were conducted using SAS version 9.4 (SAS Institute, Inc., Cary, NC, USA) and Stata version 17.0 (Stata Corp LP, College Station, TX).

We estimated survival using the cumulative incidence function, applied with the SAS macro %CIF. The survival function is defined as 1 minus the cumulative incidence function (CIF). 1-,2-, 3-, and 5-year overall survival after a colon or rectal cancer diagnosis, by spread of disease, were estimated using RBDM records. Individuals alive at the end of the follow-up (for this analysis, 30 June 2017) were censored. 1- and 2-year cancer-specific survival after diagnosis, by spread of disease, were estimated using NSWCR records. Death from other causes (i.e., codes other than C18 for colon cancer and C19-20 for rectal cancer) were competing events, censoring those alive at the end of follow-up (for this analysis, 31 December 2013).

We examined frequencies of treatment categories based on treatment received in the 0-2 years post-diagnosis (with sensitivity analyses based on the 0-1 and 0-5 years post-diagnosis, Additional file 3).

We carried out two time-to-treatment analyses based on treatment 0-2 years post-diagnosis. In the first analysis, we considered all cases with colon cancer or, separately, rectal cancer, and measured time from the date of diagnosis to date of first treatment, date of death, or end of follow-up (2 years post-diagnosis) using Fine and Gray’s competing risks cumulative incidence function (CIF) [32, 33] with the SAS macro %CIF. We used the CIF rather than the Kaplan-Meier method as the presence of competing risks may violate the assumption of non-informative censoring of the Kaplan-Meier method. The competing events were all treatment categories other than the treatment category of interest, in addition to the category “no treatment, died”. Those assigned to “no treatment, alive” were censored. In the second analysis, we considered only those cases who had a record of treatment receipt, and used the empirical cumulative distribution function (ECDF) to estimate time to first treatment receipt for these cases, by treatment category (with categories based on all treatment 0-2 years post-diagnosis). As an additional analysis, we used the ECDF to examine time to first treatment for those who received treatment, by spread of disease.

For people with “no record of cancer-related treatment, alive” at 2 years post-diagnosis, we considered records of procedures that could indicate potential CRC surveillance following definitive treatment not captured in the linked data (e.g. all tumour tissue excised through the initial biopsy). In particular, we calculated the proportion of these people who had records of computed tomography (CT) scan and/or colonoscopy ≤2 years post-diagnosis (see Additional file 2 for procedure codes) [34].

For the association analyses between treatment receipt and clinical/sociodemographic/lifestyle characteristics, we used Fine and Gray’s competing risks subdistribution hazard model and the SAS procedure PHREG with ‘eventcode’ and ‘rl’ options to obtain subdistribution hazard ratios (SHRs) and 95% confidence intervals (CIs). For each treatment category, other treatment categories and death were competing events. For the outcome “no treatment, died”, the treatment categories were the competing events. For all models, those with “no treatment, alive” at 2-years post-diagnosis were censored. Estimates were fully adjusted for all characteristics in the model. For characteristics with missing data for ≥5% of people with cancer, missing values were treated as a separate “unspecified” category. For characteristics with missing data for <5% of people with colon or rectal cancer, individuals with missing values were excluded from the association analyses. The number of people with colon and rectal cancer, by each characteristic and treatment category, is shown in Additional file 4. The proportional subdistribution hazard assumption was assessed using the STATA package STCRREG with ‘tvc’ option. Significance was defined using Bonferroni adjustment for 16 tests (i.e., *p*<0.003, accounting for 16 characteristics).

Previous studies indicate that the subdistribution hazard model is the recommended method for estimating incidence in the presence of competing risks, whereas Cox’s cause-specific hazard model is recommended when the focus is on addressing aetiological questions [32, 35]. To allow for a comprehensive understanding of all event dynamics, we additionally 1) estimated hazard ratios (HRs) and 95% CIs using separate Cox’s cause-specific hazards models for each outcome [35], and 2) used the joint Cox model [36, 37] to examine the association between each characteristic and all outcomes in the same model. For both analyses, we used the SAS procedure PHREG. Detailed descriptions of all three models are shown in Additional file 5.

## Results

We included 1,236 and 542 participants in the 45 and Up Study with a new colon and rectal cancer diagnosis in 2006-2013, respectively (Additional file 6).

People with incident colon cancer had a median age of 72 years at diagnosis (interquartile range (IQR) 65-80). 33.4% had localised spread of disease, 40.6% had regional and 20.4% had distant disease at diagnosis, similar to all people diagnosed with colon cancer in NSW and Australia during 2010-2017 (Additional file 7). 1-, 2- and 5-year overall survival was 84.2%, 76.4% and 63.7%, respectively (Additional file 8). We found substantial variation by spread of disease, e.g. 5-year overall survival was 85.3%, 68.6% and 15.9% for people with localised disease, regional and distant spread of disease, respectively. 1- and 2-year cancer-specific survival was 86.4% and 79.8%, respectively (Additional file 9).

People with rectal cancer had a median age of 68 years at diagnosis (IQR 61-77). 33.2% had localised disease and 16.4% had distant disease, similar to all people diagnosed with rectal cancer in NSW and Australia (Additional file 7), with a slightly larger proportion of people in the 45 and Up Study with regional disease (42.1% versus 32.5% for all people with incident rectal cancer in NSW). 1-, 2- and 5-year overall survival was 87.1%, 81.2% and 66.7%, respectively (Additional file 8), also with strong differences by spread of disease (e.g. 5-year overall survival was 84.0%, 71.0% and 15.7% for people with localised disease, regional and distant spread of disease, respectively). 1- and 2-year cancer-specific survival was 90.4% and 84.3%, respectively (Additional file 9).

### Receipt of cancer treatment

#### Colon cancer

**Fig. 1:**
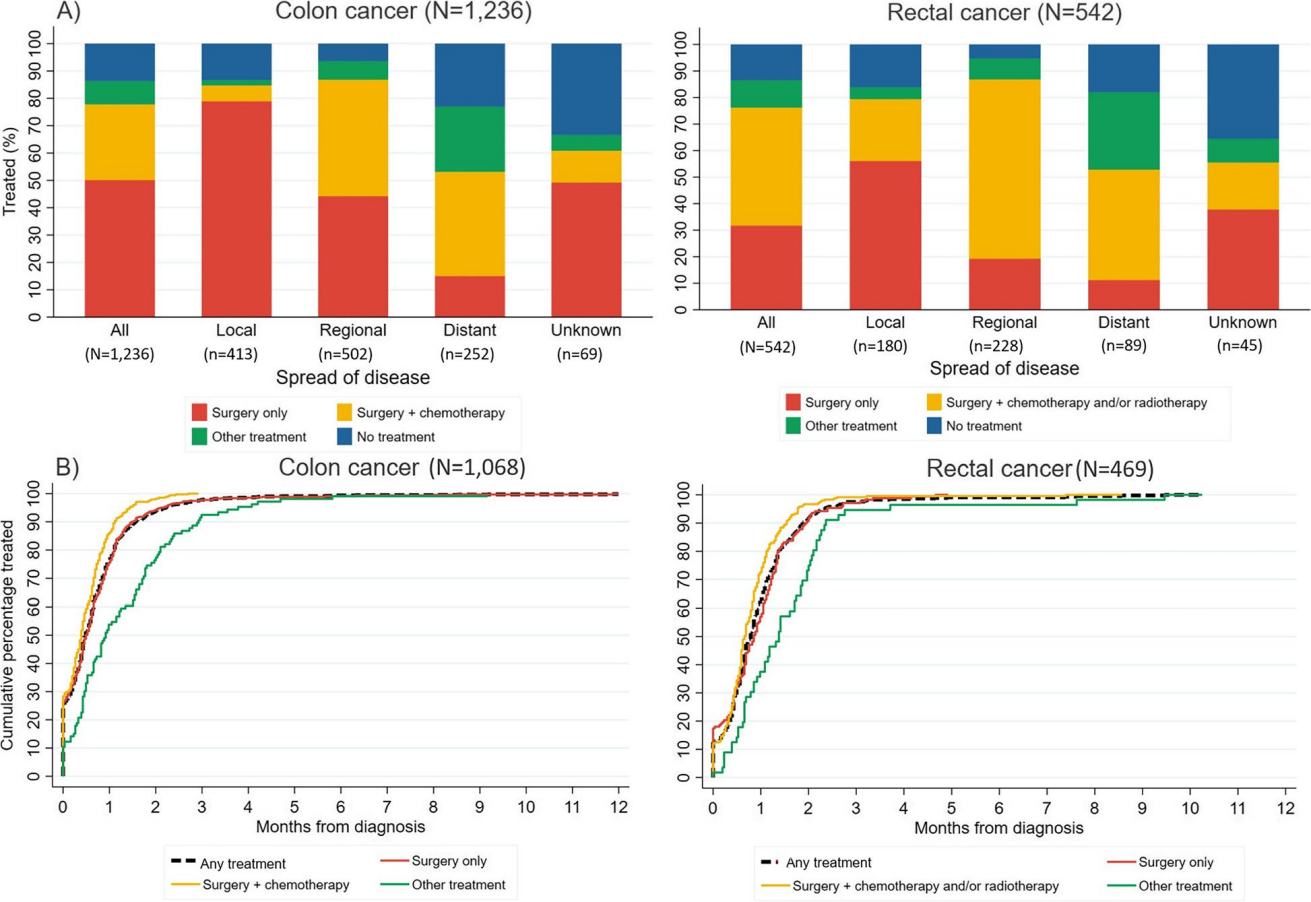
Cancer treatment received by 45 and Up Study participants with incident colon cancer (left; *n*=1,236) or rectal cancer (right; *n*=542) after baseline. **A** Treatment received in the 0-2 years after the cancer diagnosis, by spread of disease at diagnosis. Exact percentages and estimates based on the 0-1 and 0-5 years after diagnosis are provided in Additional file 3. **B** Time from diagnosis to first cancer treatment, by treatment category based on all treatment in the 0-2 years after diagnosis. The figure includes only people who received treatment within 2 years after their cancer diagnosis (*n*=1068 for colon cancer, *n*=469 for rectal cancer). The cumulative percentage was obtained using the empirical cumulative distribution function

In the 0-2 years after diagnosis, 50.2% of all people with colon cancer had “surgery only”, 27.7% “surgery plus chemotherapy”, 8.6% “other treatment”, and 6.3% “no treatment, died” (Fig. 1A and Additional file 3). 7.3% were assigned to “no treatment, alive”; of these people, 73.3% had a CT scan and/or colonoscopy in the 2 years post-diagnosis, indicating potential surveillance for CRC, possibly following definitive treatment not captured by the linked data. Treatment receipt varied greatly by spread of disease. A higher proportion of people with localised disease had “surgery only” (78.9%), compared to 44.2% and 15.1% of people with regional and distant spread of disease, respectively. A similar proportion of people with regional and distant disease had “surgery plus chemotherapy” (42.6% and 38.1%, respectively). Treatment receipt for people with distant disease was more heterogenous (15.1% had “surgery only”, 23.8% “other treatment” and n~58 (23.0%) “no treatment, died”).

86.4% of all people with colon cancer received treatment (Additional file 10). Of those treated, 75.8%, 93.3% and 97.8% received their first treatment within 1, 2 and 3 months after diagnosis, respectively (Fig. 1B). Time to treatment receipt differed by treatment category. For example, 97.7% of people who had “surgery plus chemotherapy” received their first treatment ≤2 months post-diagnosis, compared to 75.5% of people with final treatment in the “other treatment” category. Time to treatment receipt was similar by spread of disease (Additional file 11).

#### Rectal cancer

In the 0-2 years after diagnosis, 31.7% of all people with rectal cancer had “surgery only”, 44.5% “surgery plus chemotherapy and/or radiotherapy”, 10.3% “other treatment”, and 5.0% “no treatment, died” (Fig. 1A and Additional file 3). 8.5% of people with rectal cancer were assigned to “no treatment, alive”; of these, 76.1% had a CT scan and/or colonoscopy in the 2 years post-diagnosis, indicating potential surveillance for CRC (as for colon cancer above). Similar to colon cancer, the treatment received varied by spread of disease. A higher proportion of people with localised disease had “surgery only” compared to distant disease (56.1% versus 11.2%, respectively), more people with regional or distant spread disease had “surgery plus chemotherapy and/or radiotherapy” (67.5% and 41.6%, respectively), and a larger proportion of those with distant disease were assigned to “no treatment, died” (n~16 (18.0)% versus *n*<5 (≤3%) of people with localised disease and regional spread).

86.5% of all people with rectal cancer received treatment (Additional file 10). Of those treated, 61.4%, 90.8% and 97.0% received their first treatment within 1, 2 and 3 months after diagnosis, respectively (Fig. 1B). Time to treatment for rectal cancer also differed by treatment category. For example, 96.3% of people who had “surgery plus chemotherapy and/or radiotherapy” received their first treatment within 2 months after diagnosis, compared to 69.6% of people with final treatment in the “other treatment” category. Time to treatment receipt was similar by spread of disease (Additional file 11).

### Characteristics associated with treatment receipt

The association analyses included 1,149 and 499 people with colon and rectal cancer, respectively (see Methods).

#### Colon cancer

**Fig. 2:**
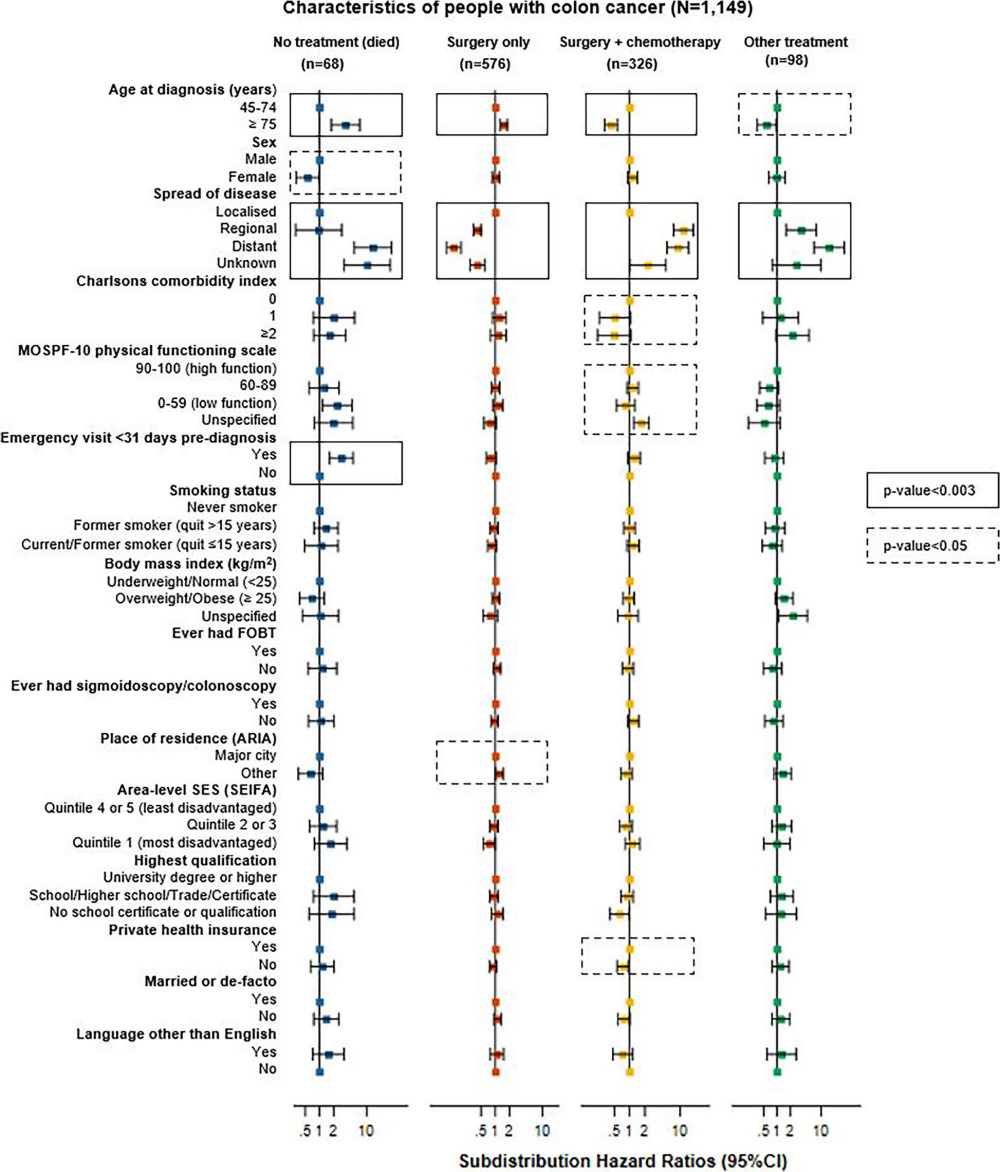
Association between characteristics of colon cancer cases and cancer treatment received in the 0-2 years after the cancer diagnosis, separately for each treatment category. Subdistribution hazard ratios were adjusted for all characteristics shown in the figure. Bars show 95% confidence intervals (95%CIs). Numbers of cases in each category are shown in Additional file 4; subdistribution hazard ratio estimates, 95%CIs and p-values are detailed in Additional file 12

Characteristics significantly associated with colon cancer treatment receipt include age at diagnosis, spread of disease and emergency presentation (all *p*≤0.003, Fig. 2 and Additional file 12; here and below, after adjusting for all other characteristics). Compared to people with colon cancer aged 45-75 years, those aged ≥75 years had a higher rate of “no treatment, died” (SHR=3.6, 95%CI:1.8-7.1) or “surgery only” (SHR=1.5, 95%CI:1.3-1.8), and a lower rate of “surgery plus chemotherapy” (SHR=0.4, 95%CI:0.3-0.6). People with regional or distant spread of disease had a higher rate of “surgery plus chemotherapy” (SHR=11.6, 95%CI:7.4-18.3 and SHR=9.0, 95%CI: 5.5-14.7, respectively) or “other treatment” (SHR=3.5, 95%CI:1.6-7.6 and SHR=15.1, 95%CI: 6.8-33.2, respectively), and a lower rate of “surgery only” (SHR=0.4, 95%CI:0.3-0.5 and SHR=0.1, 95%CI:0.1-0.2, respectively), than people with localised disease. People with distant spread of disease had a higher rate of “no treatment, died” (SHR=13.6, 95%CI:5.5-33.9) compared to those with localised disease. An emergency department visit ≤1 month pre-diagnosis was significantly associated with “no treatment, died” (SHR=2.9, 95%CI:1.6-5.2).

Characteristics with only nominally significant associations with colon cancer treatment receipt (0.003<*p*≤0.05) include sex, Charlson’s comorbidity index, level of physical functioning, place of residence and private health insurance. Females had a lower rate of “no treatment, died” (SHR=0.6, 95%CI:0.3-1.0). People with a Charlson’s comorbidity index score 1 had a lower rate of “surgery plus chemotherapy" (SHR=0.5, 95%CI: 0.3-1.0) than those with score 0. People with unspecified physical functioning (i.e. missing responses to some questionnaire items) had a higher rate of “surgery plus chemotherapy” (SHR=1.7, 95%CI:1.2-2.4) than those with high physical functioning. People with colon cancer living outside major cities had a higher rate of “surgery only” (SHR=1.2, 95%CI:1.0-1.5) and people with no private health insurance had a lower rate of “surgery plus chemotherapy” (SHR=0.7, 95%CI:0.6-0.9).

#### Rectal cancer

**Fig. 3:**
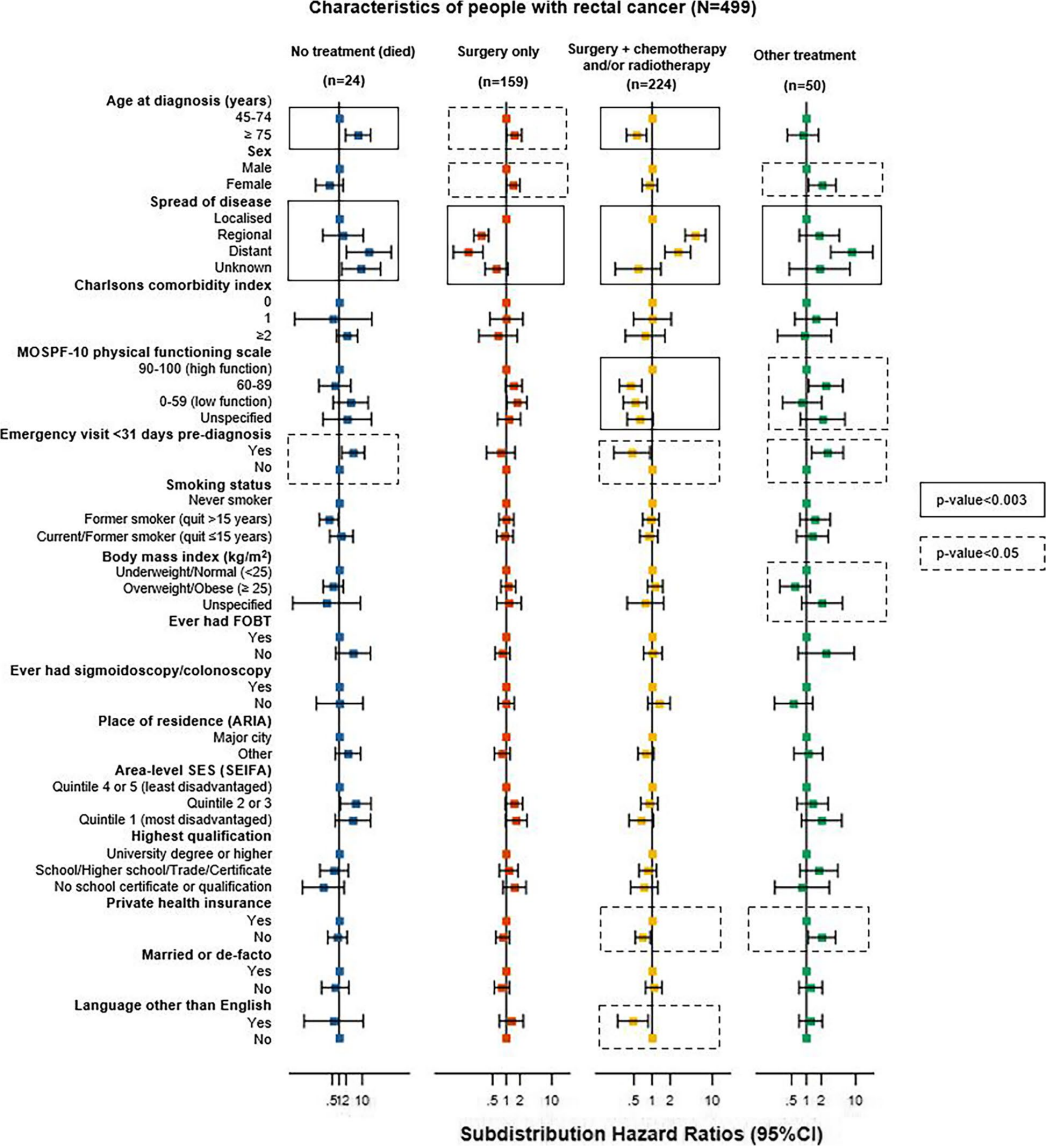
Association between characteristics of rectal cancer cases and cancer treatment received in the 0-2 years after the cancer diagnosis, separately for each treatment category. Subdistribution hazard ratios were adjusted for all characteristics shown in the figure. Bars shows 95% confidence intervals (95%CIs). Numbers of cases in each category are shown in Additional file 4; subdistribution hazard ratio estimates, 95%CIs and p-values are detailed in Additional file 13

Characteristics significantly associated with rectal cancer treatment receipt were age at diagnosis, spread of disease and level of physical functioning at baseline (all *p*≤0.003, Fig. 3 and Additional file 13). People with rectal cancer aged ≥75 years had a higher rate of “no treatment, died” (SHR=6.6, 95%CI:1.9-23.0), and lower rate of “surgery plus chemotherapy and/or radiotherapy” (SHR=0.6, 95%CI:0.4-0.8) than people aged 45-74 years. People with regional or distant spread of disease had a higher rate of “surgery plus chemotherapy and/or radiotherapy” (SHR=5.2, 95%CI:3.6-7.7 and SHR=2.7, 95%CI: 1.6-4.4, respectively) and lower rate of “surgery only” (SHR=0.3, 95%CI:0.2-0.4 and SHR=0.1, 95%CI:0.1-0.3, respectively) than people with localised disease. People with distant spread of disease had a higher rate of “no treatment, died” (SHR=19.1, 95%CI:2.1-177.4) or “other treatment” (SHR=8.4, 95%CI:3.1-22.4) than people with localised disease. People with lower physical functioning at baseline had a lower rate of “surgery plus chemotherapy and/or radiotherapy” than people with higher physical functioning (e.g., 0-59 low functioning versus 90-100 high functioning SHR=0.5, 95%CI:0.3-0.8).

Characteristics with only nominally significant associations with rectal cancer treatment receipt include sex, emergency presentation, private health insurance and language other than English spoken at home (0.003<*p*≤0.05). Females had a higher rate of “surgery only” (SHR=1.4, 95%CI:1.0-2.0). An emergency department visit ≤1 month pre-diagnosis was associated with “no treatment, died” (SHR=4.0, 95%CI:1.3-12.5), “other treatment” (SHR=2.7, 95%CI:1.3-5.6), and “surgery plus chemotherapy and/or radiotherapy” (SHR=0.5, 95%CI:0.2-0.9). People with rectal cancer and no private health insurance had a higher rate of “other treatment” (SHR=2.1, 95%CI:1.1-3.9) and lower rate of “surgery plus chemotherapy and or radiotherapy” (SHR=0.7, 95%CI:0.5-0.9). People who spoke a language other than English at home had a lower rate of “surgery plus chemotherapy and/or radiotherapy” (SHR=0.5, 95%CI:0.3-0.9).

The additional analyses using cause-specific and joint Cox hazard model indicate there was high agreement across all models on the characteristics most strongly associated with treatment receipt (*p*<0.003; Additional files 12 and 13). All three models also consistently produced effect estimates indicating the same direction of effect.

## Discussion

This study provides detailed insights into the treatment patterns of incident colon and rectal cancer cases in Australia. Our results highlight that 86.4% of colon and 86.5% of rectal cancer cases had a record of receiving treatment within 2 years post-diagnosis, and of those treated, 93.2% and 90.8% received their first treatment within 2 months after diagnosis, respectively. Characteristics significantly associated with treatment receipt were generally similar for both cancers; the strongest associations were with spread of disease and age at diagnosis.

Among 45 and Up Study participants with colon and rectal cancer, the distribution for spread of disease at diagnosis was similar to that reported for all people with colon and rectal cancer in NSW in 2013-2017 [38]. Moreover, the 5-year relative survival for Australians aged ≥50 years who were diagnosed with colorectal cancer in 2011 was 69.6% [39], broadly consistent with the 5-year overall survival estimates from this study (63.6% for colon cancer and 66.7% for rectal cancer, noting the difference in survival measures).

### Time from diagnosis to treatment

Recommendations for timelines from diagnosis to cancer treatment exist in multiple countries [17, 20]. In Australia, the optimal care pathway guidelines [17, 18, 40] suggest an optimal timeframe of up to 7-9 weeks from a colon or rectal cancer diagnosis to first treatment, broadly similar to the National Health Service UK guidelines [41]. In our study, of the colon and rectal cancer cases who received treatment, 93.2% and 90.8% had their first treatment ≤2 months post-diagnosis, respectively, indicating the majority of cases received treatment within the optimal timeframe. However, this varied by treatment category. 97.7% and 96.3% of people with colon and rectal cancer with a final category of “surgery plus chemotherapy” or “surgery plus chemotherapy and/or radiotherapy”, respectively, received treatment ≤2 months after diagnosis. By contrast, only 75.5% and 69.6% of people with colon and rectal cancer with final categories of “other treatment” received treatment in this same timeframe (though this might be partially due to “other treatment” also including mainly palliative treatments, in which case earlier care may not necessarily represent optimal care). In general, timeliness of treatment is an important factor of cancer care, and important to benchmark to assess the long-term consequences of cancer care delays and disruptions. This necessity has been highlighted by the COVID-19 pandemic and the emerging impact on cancer outcomes [42, 43].

### Characteristics associated with different treatment categories

Spread of disease was the strongest predictor of treatment received ≤2 years after diagnosis. People with localised disease had a higher rate of “surgery only”. People with distant spread of disease had a higher rate of “no treatment, died”. People with colon cancer and regional or distant spread of disease had a higher rate of “surgery plus chemotherapy” or “other treatment”, and people with rectal cancer and with regional or distant spread of disease had a higher rate of “surgery plus chemotherapy and/or radiotherapy”. These results are broadly consistent with the previous and current guidelines for the treatment of CRC [17–19, 44]. For example, for stage I disease, surgery is the main treatment, whereas there are several different treatment pathways for stage IV disease. This emphasises the complexity of treating advanced-stage disease, and the importance of early diagnosis. Detection at early stages can improve survival and lessen the demand for more complex treatment and reduce associated treatment cost [8]; in Australia, screening provided by the National Bowel Cancer Screening Program has been shown to improve CRC outcomes [45, 46].

People with colon and rectal cancer and older at diagnosis had a higher rate of no treatment record and death, an association well documented in Australia [9, 14] and worldwide [3, 5]. Colon and rectal cancer treatment decisions for older and often frailer patients are complex. Older patients are underrepresented in clinical trials, creating an evidence gap which makes it even more challenging for physicians to make optimal treatment decisions for this group [47, 48]. With ageing populations in high-income countries, it is important to address this evidence gap.

People with colon and rectal cancer and private health insurance and/or better overall health (e.g. higher physical functioning at baseline and fewer comorbidities) had a higher rate of the treatment categories “surgery plus chemotherapy” or “surgery plus chemotherapy and/or radiotherapy”, respectively. Previous research found 45 and Up Study participants with private health insurance at baseline were more likely to have higher education, be health conscious, and have higher income at baseline [49]. This may suggest different health-seeking behaviours for individuals with private health insurance. People with rectal cancer who spoke a language other than English at home were also less likely to receive “surgery plus chemotherapy and/or radiotherapy”. The combination of these factors suggest specific groups may have different access to cancer treatment services, warranting further investigation.

We also found 22.8% and 10.4% of people with colon and rectal cancer, respectively, visited an emergency department in the month prior to diagnosis; this was associated with the “no treatment, died” category. However, we cannot determine if the emergency department visit was related to the cancer diagnosis (see Additional file 14 for further discussion). Moreover, there may be interdependencies between emergency department visits and other characteristics, especially the presence of comorbidities (which in turn is also related to age). In-depth investigation of such complex relationships and their impact on treatment receipt would require large sample sizes. This was beyond the scope of the current study and could be the subject of future work.

### Implications

The estimates reported here will add to the understanding of colon and rectal cancer treatment patterns in Australia and inform future research on optimising CRC outcomes. The study findings can be used to inform the inclusion of treatment pathways in predictive models of CRC, previously used to evaluate the health outcomes and cost-effectiveness of screening and other interventions targeted at reducing the burden of colon and rectal cancer [46, 50–53]. For example, Policy1-Bowel is a comprehensive, calibrated and validated microsimulation modelling platform for colon and rectal cancer in Australia that incorporates multiple aspects of cancer control and has already informed several national policy decisions [46, 50–52].

### Limitations and strengths

Our study has some limitations. We did not have detailed TNM staging of disease and were unable to examine alignment of treatment patterns with the clinical practice guidelines, or to which extent patients’ preferences or specific clinical considerations influenced treatment decisions. Data on Eastern Cooperative Oncology Group (ECOG) performance status at diagnosis was not available. Characteristics obtained at baseline may have changed before cancer diagnosis. There is also the potential for residual confounding in the analyses examining characteristics associated with treatment receipt. The 45 and Up Study cohort does not include participants <45 years old, for whom treatment patterns may be different [54]. Cohort participants are also generally more socioeconomically advantaged, healthier and more health conscious, therefore, estimates from this study may not be entirely representative of the Australian population. The examined treatment categories were defined based on all treatment records in the 0-2 years post-diagnosis, which may have included treatment for stage progression and/or recurrence. However, sensitivity analyses based on the treatment in the 0-6 months or 0-1 years post-diagnosis showed very similar frequencies of all treatment categories (see Additional file 3). Some of the treatment categories had small sample sizes, including “no treatment, died” category; thus some hazard ratio estimates have wide confidence intervals and may not be robustly estimated. However, our study also has notable strengths, including extensive linkage to routinely collected health data, which enabled detailed insights into treatment patterns and the associations between different characteristics and treatment receipt. Our study includes a larger sample than previous Australian studies, allowing us to separately examine colon and rectal cancer. Finally, we considered different treatment combinations rather than single types of treatment, which allowed for separate examination of more complex treatment approaches, e.g. distinguishing surgery only and surgery plus chemotherapy.

## Conclusion

The characterisation of colon and rectal cancer treatment patterns and their considerable variation by spread of disease and age can help estimate future healthcare requirements with rising colon and rectal cancer burden, and help model the health and economic impacts of cancer control interventions to improve prevention and early diagnosis. The assessment of time to treatment prior to the COVID-19 pandemic can also be used as a benchmark against which to assess the extent of treatment delays and disruptions during the pandemic.

## Supplementary Information

**Additional file 1.** Diagnosis codes used to identify conditions in the Admitted Patient Data Collection and derive Charlson’s comorbidity index score. The total index score was calculated as the total sum of updated weights for conditions captured in the 5 years prior to diagnosis.

**Additional file 2.** Codes used to identify cancer treatment procedures in the different health datasets.

**Additional file 3.** Colon and rectal cancer cases assigned to each treatment category based on treatment received in the 0-1, 0-2, and 0-5 years after diagnosis, by spread of disease.

**Additional file 4.** Characteristics of colon cancer and rectal cancer cases by type of cancer treatment received in the 0-2 years after diagnosis.

**Additional file 5.** Detailed information on the subdistribution, separate Cox’s cause-specific and joint Cox hazard model and application of each method using the SAS procedure PHREG.

**Additional file 6.** Selection of colon and rectal cancer cases from the 45 and Up Study cohort for inclusion in this study.

**Additional file 7.** Age and spread of disease at diagnosis of colon and rectal cancer cases in the 45 and Up Study, and of all cases in NSW and Australia.

**Additional file 8.** Overall 1-, 2-, 3- and 5-year survival after a diagnosis of colon or rectal cancer, by spread of disease, based on RBDM data to June 2017.

**Additional file 9.** Cancer-specific 1- and 2-year survival after a diagnosis of colon or rectal cancer, by spread of disease, based on NSWCR data to 31 December 2013.

**Additional file 10.** Time from diagnosis to first cancer treatment received in the 0-2 years after diagnosis, by treatment category. The cumulative percentage was obtained using the cumulative incidence function (CIF).

**Additional file 11.** Time from diagnosis to first cancer treatment received in the 0-2 years after diagnosis, by spread of disease. The cumulative percentage was obtained using the empirical cumulative distribution function (ECDF).

**Additional file 12.** Multivariable adjusted hazard ratios for associations between characteristics of colon cancer cases and cancer treatment received within 2 years after cancer diagnosis. Subdistribution hazard ratios (SHRs) from the competing risks Fine-Gray model, and hazard ratios (HRs) from the cause-specific Cox hazard and joint Cox model were adjusted for all characteristics shown in the table. *P*-values with two asterisks (**) are significant after Bonferroni adjustment for 16 tests (i.e., *p*<0.003), while those with one asterisk (*) are significant at a nominal level of *p*<0.05.

**Additional file 13.** Multivariable adjusted hazard ratios for associations between characteristics of rectal cancer cases and treatment received within 2 years from the cancer diagnosis. SHRs from the competing risks Fine-Gray model, and HRs from the cause-specific Cox hazard and joint Cox model were adjusted for all characteristics in the table. *P*-values with two asterisks (**) are significant after Bonferroni adjustment for 16 tests (i.e., *p*<0.003), while those with one asterisk (*) are significant at a nominal level of *p*<0.05.

**Additional file 14.** Details on an emergency presentation prior to cancer diagnosis.

## Data Availability

The 45 and Up Study data that support the findings of this study are available from the Sax Institute (see https://www.saxinstitute.org.au/our-work/45-up-study/for-researchers/ for details) but restrictions apply to the availability of these data, which were used under license for the current study, and so are not publicly available. Therefore, the authors cannot on-provide the data to other researchers. However, researchers are able to access these data from the relevant data custodians for approved research projects, and enquiries for data access can be made to the Sax Institute.

## Abbreviations

APDC: Admitted Patient Data Collection
ARIA: Accessibility and Remoteness Index of Australia
CI: Confidence Interval
CIF: Cumulative Incidence Function
CRC: Colorectal Cancer
CT: Computed Tomography
ECDF: Empirical Cumulative Distribution Function
EDDC: Emergency Department Data Collection
FOBT: Faecal Occult Blood Test
MOSPF-10: Medical Outcomes Study Physical Functioning 10
NSW: New South Wales
NSWCR: New South Wales Cancer Registry
RBDM: Registry of Births, Death and Marriages
SEIFA: Socio-Economic Indexes for Areas
SES: Socio-Economic Status
SHR: Subdistribution Hazard Ratio
TNM: Tumour, Nodes, Metastases
