# Supplementary material for: Colon and rectal cancer treatment patterns and their associations with clinical, sociodemographic and lifestyle characteristics: analysis of the Australian 45 and Up Study cohort
Source: BMC Cancer. 2023 Jan 18;23:60. doi: 10.1186/s12885-023-10528-8 (PMC9845101; doi:10.1186/s12885-023-10528-8)
Supplement: Supplementary file 1 — Additional file 1. Diagnosis codes used to identify conditions in the Admitted Patient Data Collection and derive Charlson’s comorbidity index score. The total index score was calculated as the total sum of updated weights for conditions captured in the 5 years prior to diagnosis. [file 12885_2023_10528_MOESM1_ESM.docx]

**Additional file 1. Diagnosis codes used to identify conditions in the Admitted Patient Data Collection and derive Charlson’s comorbidity index score. The total index score was calculated as the total sum of updated weights for conditions captured in the 5 years prior to diagnosis.**

| **Condition** | **Diagnosis codes (ICD-10-AM)** | **Updated weight** |
| --- | --- | --- |
| Acute myocardial infarction | I21, I22, I25.2 | 0 |
| Congestive heart failure | I50 | 2 |
| Peripheral vascular disease | I71, R02, I73.9, I79.0, Z95.8, Z95.9 | 0 |
| Cerebral vascular accident | I60, I61, I62, I63, I64, I65, I66, I69, G46, G45.0, G45.1, G45.2, G45.4, G45.8, G45.9, I67.0, I67.1, I67.2, I67.4, I67.5, I67.6, I67.7, I67.8, I67.9, I68.1, I68.2, I68.8 | 0 |
| Dementia | F00, F01, F02, F05.1 | 2 |
| Pulmonary disease | J40, J41, J42, J43, J44, J45, J46, J47, J60, J61, J62, J63, J64, J65, J66, J67 | 1 |
| Connective tissue disorder | M32, M34, M05.0, M05.1, M05.2, M05.3, M05.8, M05.9, M06.0, M06.3, M06.9, M33.2, M35.3 | 1 |
| Peptic ulcer | K25, K26, K27, K28 | 0 |
| Liver disease | K73, K70.2, K70.3, K71.7, K74.0, K74.2, K74.3, K74.4, K74.5, K74.6 | 2 |
| Diabetes without complications | E10.9, E11.9, E13.9, E14.9, E10.1, E11.1, E13.1, E14.1, E10.5, E11.5, E13.5, E14.5 | 0 |
| Diabetes with complications | E10.2, E11.2, E13.2, E14.2, E10.3, E11.3, E13.3, E14.3, E10.4, E11.4, E13.4, E14.4 | 1 |
| Paraplegia | G81, G04.1, G82.0, G82.1, G82.2 | 2 |
| Renal disease | N05.2, N05.3, N05.4, N05.5, N05.6, N07.2, N07.3, N07.4, N03, N01, N18, N19, N25 | 1 |
| Severe liver disease | K72.9, K76.6, K76.7, K72.1 | 4 |
| AIDS/HIV | B20, B21, B22, B23, B24 | 4 |
