# Supplementary material for: Colon and rectal cancer treatment patterns and their associations with clinical, sociodemographic and lifestyle characteristics: analysis of the Australian 45 and Up Study cohort
Source: BMC Cancer. 2023 Jan 18;23:60. doi: 10.1186/s12885-023-10528-8 (PMC9845101; doi:10.1186/s12885-023-10528-8)
Supplement: Supplementary file 2 — Additional file 2. Codes used to identify cancer treatment procedures in the different health datasets. [file 12885_2023_10528_MOESM2_ESM.docx]

**Additional file 2. Codes used to identify cancer treatment procedures in the different health datasets**

| **Procedure** | **Data type** | **Codes** |
| --- | --- | --- |
| Surgery | APDC procedure (including all sub-codes) | 32000, 32003-32015, 32024-32026, 32028, 32030, 32033, 32039, 32042, 32045-32047, 32051, 32099, 32102-32106, 32108, 32112, 90308, 90315, 90341, 90450, 90959 |
|  | MBS item | 32000, 32003-32015, 32024-32026, 32028, 32030, 32033, 32039, 32042, 32045-32047, 32051, 32099, 32102-32106, 32108, 32112 |
| Chemotherapy | APDC procedure (including all sub-codes) | 13915-00, 13918-00, 13921-00, 13924-00, 13927-00, 15304, 15312, 15319, 15327, 15338, 15360, 90760-00, 90767-00, 90768-00, 96199-00, 96199-09, 96200-00, 96201-00, 96203-00, 96204-00 |
|  | APDC diagnosis | Z51.1, Z51.2 |
|  | MBS item | 13915, 13918, 13921, 13924, 13927, 13930, 13933, 13936, 13945 |
|  | PBS ATC code | All codes starting with “L01” (L01AA01-L01XX41) excluding L01BA01 |
| Radiotherapy | APDC procedure | 15100-00,15203-00,15204-00,15207-00,15208-00,15224-00,15239-00, 15254-00, 15269-00, 15506-01, 15506-02, 15518-00, 15521-00, 15524-00, 15550-00, 15600-00, 15600-01, 90765-00, 90765-01, 90765-02,90765-03 |
|  | APDC diagnosis | Z51.0 |
|  | MBS item | 15000-15399, 15500-15600, 15700-15899 |
| Colonoscopy | MBS item | 32084, 32087, 32088, 32089, 32090, 32093, 32094, 32095, 32222, 32223, 32224, 32225, 32226, 32227, 32228, 32229 |
| Computed Tomography scan | MBS item | 56401, 56407, 56501, 56507, 56553, 56801, 56807, 56441, 56507, 56552, 56554 |
