# Supplementary material for: Colon and rectal cancer treatment patterns and their associations with clinical, sociodemographic and lifestyle characteristics: analysis of the Australian 45 and Up Study cohort
Source: BMC Cancer. 2023 Jan 18;23:60. doi: 10.1186/s12885-023-10528-8 (PMC9845101; doi:10.1186/s12885-023-10528-8)
Supplement: Supplementary file 3 — Additional file 3. Colon and rectal cancer cases assigned to each treatment category based on treatment received in the 0-1, 0-2, and 0-5 years after diagnosis, by spread of disease. [file 12885_2023_10528_MOESM3_ESM.docx]

**Additional file 3. Colon and rectal cancer cases assigned to each treatment category based on treatment received in the 0-1, 0-2, and 0-5 years after diagnosis, by spread of disease.**

The estimates for the 0-1 and 0-2 years after diagnosis are based on all incident colon and rectal cancer cases in the 45 and Up Study (N=1,236 and N=542, respectively), while the estimates for the 0-5 years after diagnosis only includes colon and rectal cancer cases with follow-up data for 5+ years (N=598 and N=278, respectively).

| **Treatment received** | **0-1 year after diagnosis** | | |  |  | **0-2 years after diagnosis** | | |  |  | **0-5 years after diagnosis** | | |  |  |
| --- | --- | --- | --- | --- | --- | --- | --- | --- | --- | --- | --- | --- | --- | --- | --- |
|  | **all  (N=1236, 100%)** | **localised  (n=413, 33.4%)** | **regional  (n=502, 40.6%)** | **distant  (n=252, 20.4%)** | **unknown  (n=69, 5.6%)** | **all  (N=1236, 100%)** | **localised (n=413, 33.4%)** | **regional  (n=502, 40.6%)** | **distant  (n=252, 20.4%)** | **unknown  (n=69, 5.6%)** | **all  (N=598, 100%)** | **localised (n=206, 34.4%)** | **regional  (n=219, 36.6%)** | **distant  (n=135, 22.6%)** | **unknown  (n=38, 6.4%)** |
| **Colon cancer** |  |  |  |  |  |  |  |  |  |  |  |  |  |  |  |
| Surgery only | 638 (51.6) | 330 (79.9) | 234 (46.6) | 40 (15.9) | 34 (49.3) | 620 (50.2) | 326 (78.9) | 222 (44.2) | 38 (15.1) | 34 (49.3) | 286 (47.8) | 163 (79.1) | 87 (39.7) | 21 (15.6) | 15 (39.5) |
| Surgery plus chemotherapy | 334 (27.0) | 21 (5.1) | 206 (41.0) | 99 (39.3) | 8 (11.6) | 342 (27.7) | 24 (5.8) | 214 (42.6) | 96 (38.1) | 8 (11.6) | 177 (29.6) | 15 (7.3) | 102 (46.6) | 56 (41.5) | < 5* |
| Other treatment | 94 (7.6) | 7 (1.7) | 28 (5.6) | 55 (21.8) | < 5* | 106 (8.6) | < 5* | 34 (6.8) | 60 (23.8) | < 5* | 68 (11.4) | 5 (2.4) | 20 (9.1) | 38 (28.1) | 5 (13.2) |
| No treatment (died) | 71 (5.7) | < 5* | 7 (1.4) | 52 (20.6) | < 5* | 78 (6.3) | < 5* | 10 (2.0) | ~58 (23.0)^1^ | < 5* | 34 (5.7) | 5 (2.4) | < 5* | ~20 (14.8)^1^ | < 5* |
| No treatment (alive) | 99 (8.0) | ~55 (13.3) ^1^ | 27 (5.4) | 6 (2.4) | 15 (21.7) | 90 (7.3) | 49 (11.9) | 22 (4.4) | < 5* | < 5* | 33 (5.5) | 18 (8.7) | < 5* | < 5* | < 5* |
| **Rectal cancer** | **all  (N=542, 100%)** | **localised  (n=180, 33.2%)** | **regional  (n=228, 42.1%)** | **distant  (n=89, 16.4%)** | **unknown  (n=45, 8.3%)** | **all  (N=542, 100%)** | **localised (n=180, 33.2%)** | **regional  (n=228, 42.1%)** | **distant  (n=89, 16.4%)** | **unknown  (n=45, 8.3%)** | **all  (N=278, 100%)** | **localised (n=86, 30.9%)** | **regional  (n=115, 41.4%)** | **distant  (n=51, 18.3%)** | **unknown  (n=26, 9.4%)** |
| Surgery only | 180 (33.2) | 105 (58.3) | 48 (21.1) | 10 (11.2) | 17 (37.8) | 172 (31.7) | 101 (56.1) | 44 (19.3) | 10 (11.2) | 17 (37.8) | 84 (30.2) | 43 (50.0) | 22 (19.1) | 7 (13.7) | 12 (46.2) |
| Surgery plus chemotherapy and/or radiotherapy | 232 (42.8) | 38 (21.1) | 150 (65.8) | 36 (40.4) | 8 (17.8) | 241 (44.5) | 42 (23.3) | 154 (67.5) | 37 (41.6) | 8 (17.8) | 132 (47.5) | ~26 (30.2)^1^ | 81 (70.4) | 23 (45.1) | < 5* |
| Other treatment | 57 (10.5) | 8 (4.4) | 18 (7.9) | 27 (30.3) | < 5* | 56 (10.3) | < 5* | 18 (7.9) | 26 (29.2) | < 5* | 25 (9.0) | < 5* | 5 (4.3) | 13 (25.5) | < 5* |
| No treatment (died) | 23 (4.2) | < 5* | < 5* | ~16 (17.9)^1^ | < 5* | 27 (5.0) | < 5* | < 5* | ~16 (18.0)^1^ | < 5* | 15 (5.4) | < 5* | < 5* | ~8 (15.7)^1^ | < 5* |
| No treatment (alive) | 50 (9.2) | ~29 (16.1) ^1^ | ~12 (5.3)^1^ | < 5* | 10 (22.2) | 46 (8.5) | 27 (15.0) | < 5* | < 5* | 10 (22.2) | 22 (7.9) | 13 (15.1) | < 5* | < 5* | 5 (19.2) |

* Cell sizes <5 have been suppressed, and neighbouring cells suppressed as well or rounded

^1^ Rounded to preserve confidentiality of other entries
