# Supplementary material for: Colon and rectal cancer treatment patterns and their associations with clinical, sociodemographic and lifestyle characteristics: analysis of the Australian 45 and Up Study cohort
Source: BMC Cancer. 2023 Jan 18;23:60. doi: 10.1186/s12885-023-10528-8 (PMC9845101; doi:10.1186/s12885-023-10528-8)
Supplement: Supplementary file 4 — Additional file 4. Characteristics of colon cancer and rectal cancer cases by type of cancer treatment received in the 0-2 years after diagnosis. [file 12885_2023_10528_MOESM4_ESM.docx]

**Additional file 4. Characteristics of colon cancer and rectal cancer cases by type of cancer treatment received in the 0-2 years after diagnosis.**

| **Characteristics** | **Colon cancer (N=1149)** | | | | | | **Rectal cancer (N=499)** | | | | | | |
| --- | --- | --- | --- | --- | --- | --- | --- | --- | --- | --- | --- | --- | --- |
|  | **All colon cases (N=1149, 100%)** | **Surgery only (n=576, 50.1%)** | **Surgery plus chemotherapy (n=326, 28.4%)** | **Other treatment (n=98, 8.5%)** | **No treatment, died (n=68, 5.9%)** | **No treatment, alive (n=81, 7.1%)** | **All rectal cases (N=499, 100%)** | **Surgery only (n=159, 31.9%)** | **Surgery plus chemotherapy and/or radiotherapy (n=224, 44.9%)** | **Other treatment (n=50, 10.0%)** | **No treatment, died (n=24, 4.8%)** | **No treatment, alive (n=42, 8.4%)** |  |
|  | **n (%)** | **n (%)** | **n (%)** | **n (%)** | **n (%)** | **n (%)** | **n (%)** | **n (%)** | **n (%)** | **n (%)** | **n (%)** | **n (%)** |  |
| **Age at diagnosis (years)** |  |  |  |  |  |  |  |  |  |  |  |  |  |
| 45-74 | 681 (59.3) | 308 (53.5) | 243 (74.5) | 68 (69.4) | 18 (26.5) | 44 (54.3) | 355 (71.1) | 106 (66.7) | 182 (81.3) | 30 (60.0) | 6 (25.0) | 31 (73.8) |  |
| 75+ | 468 (40.7) | 268 (46.5) | 83 (25.5) | 30 (30.6) | 50 (73.5) | 37 (45.7) | 144 (28.9) | 53 (33.3) | 42 (18.8) | 20 (40.0) | 18 (75.0) | 11 (26.2) |  |
| **Spread of disease** |  |  |  |  |  |  |  |  |  |  |  |  |  |
| Localised | 387 (33.7) | 308 (53.5) | 21 (6.4) | 8 (8.2) | 5 (7.4) | 45 (55.6) | 169 (33.9) | 93 (58.5) | 41 (18.3) | ~8 (16.0)^1^ | < 5* | 26 (61.9) |  |
| Regional | 466 (40.6) | 201 (34.9) | 208 (63.8) | 31 (31.6) | 8 (11.8) | 18 (22.2) | 214 (42.9) | 42 (26.4) | 145 (64.7) | –^1^ | < 5* | 7 (16.7) |  |
| Distant | 232 (20.2) | 37 (6.4) | 89 (27.3) | ~58 (59.1)^1^ | 48 (70.6) | < 5* | 79 (15.8) | 9 (5.7) | 32 (14.3) | 24 (48.0) | 14 (58.3) | 0 (0.0) |  |
| Unknown | 64 (5.6) | 30 (5.2) | 8 (2.5) | < 5* | 7 (10.3) | –^1^ | 37 (7.4) | 15 (9.4) | 6 (2.7) | < 5* | < 5* | 9 (21.4) |  |
| **Charlsons comorbidity index** |  |  |  |  |  |  |  |  |  |  |  |  |  |
| 0 | 1,023 (89.0) | 501 (87.0) | 311 (95.4) | 85 (86.7) | 51 (75.0) | 75 (92.6) | 452 (90.6) | 145 (91.2) | 210 (93.8) | 43 (86.0) | 16 (66.7) | 38 (90.5) |  |
| 1 | 62 (5.4) | 39 (6.8) | 8 (2.5) | –^1^ | 6 (8.8) | < 5* | 24 (4.8) | 8 (5.0) | 8 (3.6) | < 5* | < 5* | < 5* |  |
| 2+ | 64 (5.6) | 36 (6.3) | 7 (2.1) | –^1^ | 11 (16.2) | < 5* | 23 (4.6) | 6 (3.8) | 6 (2.7) | < 5* | –^1^ | < 5* |  |
| **MOSPF-10 physical functioning scale** |  |  |  |  |  |  |  |  |  |  |  |  |  |
| 90-100 (high function) | 516 (44.9) | 250 (43.4) | 163 (50.0) | 51 (52.0) | 17 (25.0) | 35 (43.2) | 270 (54.1) | 73 (45.9) | 150 (67.0) | 19 (38.0) | 7 (29.2) | 21 (50.0) |  |
| 60-89 | 311 (27.1) | 155 (26.9) | 90 (27.6) | 24 (24.5) | 16 (23.5) | 26 (32.1) | 103 (20.6) | 41 (25.8) | 31 (13.8) | 17 (34.0) | < 5* | –^1^ |  |
| 0-59 (low function) | 205 (17.8) | 116 (20.1) | 34 (10.4) | 17 (17.3) | 26 (38.2) | 12 (14.8) | 81 (16.2) | 31 (19.5) | 27 (12.1) | 8 (16.0) | 9 (37.5) | 6 (14.3) |  |
| Unspecified | 117 (10.2) | 55 (9.5) | 39 (12.0) | 6 (6.1) | 9 (13.2) | 8 (9.9) | 45 (9.0) | 14 (8.8) | 16 (7.1) | 6 (12.0) | < 5* | –^1^ |  |
| **Emergency visit <31 days pre-diagnosis** |  |  |  |  |  |  |  |  |  |  |  |  |  |
| Yes | 262 (22.8) | 93 (16.1) | 79 (24.2) | 31 (31.6) | 44 (64.7) | 15 (18.5) | 52 (10.4) | 12 (7.5) | 12 (5.4) | 14 (28.0) | –^1^ | < 5* |  |
| No | 887 (77.2) | 483 (83.9) | 247 (75.8) | 67 (68.4) | 24 (35.3) | 66 (81.5) | 447 (89.6) | 147 (92.5) | 212 (94.6) | 36 (72.0) | –^1^ | ~42 (100.0)^1^ |  |
| **Smoking status** |  |  |  |  |  |  |  |  |  |  |  |  |  |
| Never smoker | 609 (53.0) | 313 (54.3) | 169 (51.8) | 52 (53.1) | 34 (50.0) | 41 (50.6) | 223 (44.7) | 71 (44.7) | 104 (46.4) | 21 (42.0) | 11 (45.8) | 16 (38.1) |  |
| Former smoker (quit >15 years) | 297 (25.8) | 149 (25.9) | 83 (25.5) | 25 (25.5) | 18 (26.5) | 22 (27.2) | 155 (31.1) | 51 (32.1) | 67 (29.9) | 18 (36.0) | 6 (25.0) | 13 (31.0) |  |
| Current/Former smoker (quit ≤15 years) | 243 (21.1) | 114 (19.8) | 74 (22.7) | 21 (21.4) | 16 (23.5) | 18 (22.2) | 121 (24.2) | 37 (23.3) | 53 (23.7) | 11 (22.0) | 7 (29.2) | 13 (31.0) |  |
| **Body Mass Index (kg/m^2^)** |  |  |  |  |  |  |  |  |  |  |  |  |  |
| Underweight/Normal (<25) | 405 (35.2) | 203 (35.2) | 114 (35.0) | 27 (27.6) | 28 (41.2) | 33 (40.7) | 162 (32.5) | 46 (28.9) | 67 (29.9) | 20 (40.0) | 13 (54.2) | 16 (38.1) |  |
| Overweight/Obese (≥25) | 659 (57.4) | 336 (58.3) | 187 (57.4) | 61 (62.2) | 34 (50.0) | 41 (50.6) | 303 (60.7) | 98 (61.6) | 146 (65.2) | 25 (50.0) | 10 (41.7) | 24 (57.1) |  |
| Unspecified | 85 (7.4) | 37 (6.4) | 25 (7.7) | 10 (10.2) | 6 (8.8) | 7 (8.6) | 34 (6.8) | 15 (9.4) | 11 (4.9) | 5 (10.0) | 1 (4.2) | 2 (4.8) |  |
| **Ever had FOBT** |  |  |  |  |  |  |  |  |  |  |  |  |  |
| Yes | 282 (24.5) | 149 (25.9) | 81 (24.8) | 26 (26.5) | 11 (16.2) | 15 (18.5) | 98 (19.6) | 41 (25.8) | 37 (16.5) | < 5* | < 5* | 15 (35.7) |  |
| No | 867 (75.5) | 427 (74.1) | 245 (75.2) | 72 (73.5) | 57 (83.8) | 66 (81.5) | 401 (80.4) | 118 (74.2) | 187 (83.5) | ~50 (100.0)^1^ | ~24 (100.0)^1^ | 27 (64.3) |  |
| **Ever had sigmoidoscopy/colonoscopy** |  |  |  |  |  |  |  |  |  |  |  |  |  |
| Yes | 341 (29.7) | 191 (33.2) | 82 (25.2) | 29 (29.6) | 17 (25.0) | 22 (27.2) | 75 (15.0) | 30 (18.9) | 27 (12.1) | 6 (12.0) | < 5* | –^1^ |  |
| No | 808 (70.3) | 385 (66.8) | 244 (74.8) | 69 (70.4) | 51 (75.0) | 59 (72.8) | 424 (85.0) | 129 (81.1) | 197 (87.9) | 44 (88.0) | ~24 (100.0)^1^ | –^1^ |  |
| **Sex** |  |  |  |  |  |  |  |  |  |  |  |  |  |
| Male | 550 (47.9) | 279 (48.4) | 147 (45.1) | 47 (48.0) | 36 (52.9) | 41 (50.6) | 327 (65.5) | 97 (61.0) | 156 (69.6) | 27 (54.0) | 18 (75.0) | 29 (69.0) |  |
| Female | 599 (52.1) | 297 (51.6) | 179 (54.9) | 51 (52.0) | 32 (47.1) | 40 (49.4) | 172 (34.5) | 62 (39.0) | 68 (30.4) | 23 (46.0) | 6 (25.0) | 13 (31.0) |  |
| **Place of residence** |  |  |  |  |  |  |  |  |  |  |  |  |  |
| Major City | 588 (51.2) | 290 (50.3) | 172 (52.8) | 43 (43.9) | 38 (55.9) | 45 (55.6) | 227 (45.5) | 70 (44.0) | 107 (47.8) | 20 (40.0) | 7 (29.2) | 23 (54.8) |  |
| Other | 561 (48.8) | 286 (49.7) | 154 (47.2) | 55 (56.1) | 30 (44.1) | 36 (44.4) | 272 (54.5) | 89 (56.0) | 117 (52.2) | 30 (60.0) | 17 (70.8) | 19 (45.2) |  |
| **Area-level SES** |  |  |  |  |  |  |  |  |  |  |  |  |  |
| Quintile 4 or 5 (least disadvantaged) | 368 (32.0) | 191 (33.2) | 112 (34.4) | 24 (24.5) | 14 (20.6) | 27 (33.3) | 152 (30.5) | 40 (25.2) | 76 (33.9) | 11 (22.0) | 5 (20.8) | 20 (47.6) |  |
| Quintile 2 or 3 | 571 (49.7) | 296 (51.4) | 147 (45.1) | 55 (56.1) | 35 (51.5) | 38 (46.9) | 258 (51.7) | 87 (54.7) | 117 (52.2) | 25 (50.0) | 13 (54.2) | 16 (38.1) |  |
| Quintile 1 (most disadvantaged) | 210 (18.3) | 89 (15.5) | 67 (20.6) | 19 (19.4) | 19 (27.9) | 16 (19.8) | 89 (17.8) | 32 (20.1) | 31 (13.8) | 14 (28.0) | 6 (25.0) | 6 (14.3) |  |
| **Highest qualification** |  |  |  |  |  |  |  |  |  |  |  |  |  |
| University degree or higher | 195 (17.0) | 96 (16.7) | 71 (21.8) | 14 (14.3) | < 5* | –^1^ | 99 (19.8) | 26 (16.4) | 53 (23.7) | 6 (12.0) | < 5* | –^1^ |  |
| School/Higher school/Trade/Cert | 770 (67.0) | 380 (66.0) | 219 (67.2) | 69 (70.4) | 47 (69.1) | 55 (67.9) | 325 (65.1) | 102 (64.2) | 146 (65.2) | 36 (72.0) | 14 (58.3) | 27 (64.3) |  |
| No school certificate or qualification | 184 (16.0) | 100 (17.4) | 36 (11.0) | 15 (15.3) | ~19 (27.9) | –^1^ | 75 (15.0) | 31 (19.5) | 25 (11.2) | 8 (16.0) | –^1^ | –^1^ |  |
| **Private health insurance** |  |  |  |  |  |  |  |  |  |  |  |  |  |
| Yes | 680 (59.2) | 338 (58.7) | 221 (67.8) | 52 (53.1) | 28 (41.2) | 41 (50.6) | 293 (58.7) | 94 (59.1) | 149 (66.5) | 17 (34.0) | 10 (41.7) | 23 (54.8) |  |
| No | 469 (40.8) | 238 (41.3) | 105 (32.2) | 46 (46.9) | 40 (58.8) | 40 (49.4) | 206 (41.3) | 65 (40.9) | 75 (33.5) | 33 (66.0) | 14 (58.3) | 19 (45.2) |  |
| **Married or de-facto** |  |  |  |  |  |  |  |  |  |  |  |  |  |
| Yes | 799 (69.5) | 389 (67.5) | 250 (76.7) | 66 (67.3) | 40 (58.8) | 54 (66.7) | 345 (69.1) | 111 (69.8) | 162 (72.3) | 29 (58.0) | 14 (58.3) | 29 (69.0) |  |
| No | 350 (30.5) | 187 (32.5) | 76 (23.3) | 32 (32.7) | 28 (41.2) | 27 (33.3) | 154 (30.9) | 48 (30.2) | 62 (27.7) | 21 (42.0) | 10 (41.7) | 13 (31.0) |  |
| **Language other than English** |  |  |  |  |  |  |  |  |  |  |  |  |  |
| Yes | 83 (7.2) | 41 (7.1) | 20 (6.1) | 8 (8.2) | 9 (13.2) | 5 (6.2) | 46 (9.2) | 17 (10.7) | 14 (6.3) | –^1^ | < 5* | 7 (16.7) |  |
| No | 1,066 (92.8) | 535 (92.9) | 306 (93.9) | 90 (91.8) | 59 (86.8) | 76 (93.8) | 453 (90.8) | 142 (89.3) | 210 (93.8) | ~50 (100.0)^1^ | ~24 (100.0)^1^ | 35 (83.3) |  |

* Cell sizes <5 have been suppressed to preserve confidentiality

^1^ Rounded or suppressed to preserve confidentiality of other entries
