# Supplementary material for: Colon and rectal cancer treatment patterns and their associations with clinical, sociodemographic and lifestyle characteristics: analysis of the Australian 45 and Up Study cohort
Source: BMC Cancer. 2023 Jan 18;23:60. doi: 10.1186/s12885-023-10528-8 (PMC9845101; doi:10.1186/s12885-023-10528-8)
Supplement: Supplementary file 5 — Additional file 5. Detailed information on the subdistribution, separate Cox’s cause-specific and joint Cox hazard model and application of each method using the SAS procedure PHREG. [file 12885_2023_10528_MOESM5_ESM.docx]

**Additional file 5. Detailed information on the subdistribution, separate Cox’s cause-specific and joint Cox hazard model and application of each method using the SAS procedure PHREG**

We considered three different hazard models to examine the association between characteristics of colon and rectal cancer cases and types of cancer treatment received: Fine and Gray’s competing risks subdistribution hazard model [1], the Cox cause-specific hazard model, and the joint Cox hazard model [1-5].

The subdistribution hazard model includes individuals who have experienced the competing event (here, death or the start of treatment in a different final treatment category) in the risk set. This model is therefore well-suited to estimate real-world risks. However, associations between individuals’ characteristics and the event of interest in this model are also influenced by the strength of associations between the characteristics and the competing events [5].

By contrast, the cause-specific hazards model only includes individuals who have not experienced any events in the risk set. The associations between individuals’ characteristics and an event of interest are thus not subject to change when the associations between the characteristics and competing events change. The cause-specific hazards model is therefore more suited to provide aetiological insights into the event of interest. As the cause-specific hazards model can only estimate associations for each outcome separately, the strength of associations for specific characteristics cannot be directly compared across outcomes. By contrast, the joint Cox hazard model is also cause-specific, but includes multiple outcomes in the same model.

To allow for a comprehensive understanding of all event dynamics, we used the subdistribution hazards models in the main analysis to examine the association between characteristics of people with colon and rectal cancer and the cancer treatment received, with additional analyses using the cause-specific hazard model and joint Cox model. We estimated hazard ratios (HRs) and 95% CIs for all models using the SAS procedure *PHREG*. Briefly, we outline the application of each method to data from the 45 and Up Study sample.

For the subdistribution hazard model, the *eventcode* and *rl* options needs to be specified in *PHREG* to obtain subdistribution hazard ratios and 95% CIs for each treatment category outcome [6]. We show an example application to newly diagnosed colon cancer cases in the 45 and Up Study, focusing on the treatment category “surgery only” as the outcome of interest. Other treatment categories and the category “no treatment, died” were considered competing events. Those with no treatment and alive at the end of the follow-up period (here, 2 years post-diagnosis; “no treatment, alive” category) were censored. Estimates were fully adjusted for all characteristics in the model.

Pseudo-code for analyses

** Dataset name: “coloncancer_dataset”;

**Variable name: “colontrt”. All treatment category outcomes for colon cases coded as; 1=surgery only, 2=surgery plus chemotherapy, 3=other treatment, 4=no treatment, died and 5=no treatment, alive;

**Variable name: “timetofirsttrt”. Time from date of diagnosis to whichever event occurred first: treatment, death or end of follow-up (2 years post-diagnosis);

**Variable names for each characteristic in the model: “agediagnosis”, “sex”, “spreaddisease”, “charlson”, “aria”, “insurance”, “qualification”, “seifa”, “bmi”, “smoking”, “language”, “married”, “mospf10”, “emergency”, “fobt”, “sigmoidcolonoscopy”. For each variable, specify the reference category in the class statement;

** For variable “colontrt”, specify treatment category 5=no treatment, alive as censored (i.e. colontrt(5)) and treatment category 1=surgery only as the event of interest (i.e., eventcode=1)) in the model statement;

proc phreg data=coloncancer_dataset plots(overlay=stratum)=cif;

class agediagnosis(ref=’45-74’) sex(ref=’Male’) spreaddisease(ref=’Localised’) charlson(ref=’0’) aria(ref=’Major Cities’) insurance(ref=’Yes’) qualification(ref=’University degree or higher’) seifa(ref=’Quintile 1 or 2 (least)’) bmi(ref=’Underweight/Normal’) smoking(ref=’Never’) language(ref=’No’) married(ref=’Yes’) mospf10(ref=’90-100 high function’) emergency(ref=’No’) fobt(ref=’Yes’) sigmoidcolonoscopy(ref=’Yes’) / order=internal;

model timetofirsttrt*colontrt(5)=agediagnosis sex spreaddisease charlson aria insurance qualification seifa bmi smoking language married mospf10 emergency fobt sigmoidcolonoscopy / rl eventcode=1;

ods output parameterestimates=hr;

ods output modelanova=pvalue;

run;

To apply the subdistribution hazard model in STATA, using the same example as above [7]:

use coloncancer_dataset.dta

stset timetofirsttrt, failure(colontrt==1)

stcrreg i.agediagnosis i.sex i.spreaddisease i.charlson i.aria i.insurance i.qualification i.seifa i.bmi i.smoking i.language i.married i.mospf10 i.emergency i.fobt i.sigmoidcolonoscopy, compete(colontrt==2 3 4) nolog

To test the proportional subdistribution hazard assumption for each covariate, add the *tvc* function to the STATA *stcrreg* statement:

stcrreg i.agediagnosis i.sex i.spreaddisease i.charlson i.aria i.insurance i.qualification i.seifa i.bmi i.smoking i.language i.married i.mospf10 i.emergency i.fobt i.sigmoidcolonoscopy, compete(colontrt==2 3 4) tvc (agediagnosis sex spreaddisease charlson aria insurance qualification seifa bmi smoking language married mospf10 emergency fobt sigmoidcolonoscopy) noshr

To apply the cause-specific hazard model for the same example using the *PHREG* procedure in SAS, we do not use the *eventcode* option.

*For variable “colontrt” treatment categories 5, 2, 3 and 4 are censored;

proc phreg data=coloncancer_dataset;

class agediagnosis(ref=’45-74’) sex(ref=’Male’) spreaddisease(ref=’Localised’) charlson(ref=’0’) aria(ref=’Major Cities’) insurance(ref=’Yes’) qualification(ref=’University degree or higher’) seifa(ref=’Quintile 1 or 2 (least)’) bmi(ref=’Underweight/Normal’) smoking(ref=’Never’) language(ref=’No’) married(ref=’Yes’) mospf10(ref=’90-100 high function’) emergency(ref=’No’) fobt(ref=’Yes’) sigmoidcolonoscopy(ref=’Yes’) / order=internal;

model timetofirsttrt*colontrt(5 2 3 4)=agediagnosis sex spreaddisease charlson aria insurance qualification seifa bmi smoking language married mospf10 emergency fobt sigmoidcolonoscopy /rl;

ods output parameterestimates=hr;

ods output modelanova=pvalue;

run;

Finally, to apply the joint Cox model, the data were augmented using a duplication method [3, 4]. Individual records were duplicated 4 times for 4 competing events (i.e., using the same example: “surgery only”, “surgery plus chemotherapy”, “other treatment”, and “no treatment, died”). We stratified by treatment type using the *strata* option. To account for duplication of individual records, we used the option *id*. An interaction term between the stratification variable and each characteristic was added to the model. We created a binary outcome indicating whether individuals experienced an event or not. An individual could experience an event once, with all other records treated as censored. For cases that had no treatment and were alive at the end of follow-up (i.e. 2 years after diagnosis; “no treatment, alive” category), all four records were treated as censored. The below SAS code demonstrates this process.

Step 1: Augment data by duplicating observations for each outcome of interest:

** Dataset name: “coloncancer_surgery”, which is a duplicate of the dataset “coloncancer_dataset”;

** Create a variable named: “strata”, where strata=1.

** Create a variable named: “event”, 1= surgery only or 0=did not have surgery only;

data coloncancer_surgery;

set coloncancer_dataset;

if colontrt=1 then do;

strata=1; event=1;

end;

else do;

strata=1; event=0;

end;

run;

** Dataset name: “coloncancer_surgchemo”, which is a duplicate of the dataset “coloncancer_dataset”;

** Create a variable named: “strata”, where strata=2.

** Create a variable named: “event”, 1=surgery plus chemotherapy or 0=did NOT have surgery plus chemotherapy;

data coloncancer_surgchemo;

set coloncancer_dataset;

if colontrt=2 then do;

strata=2; event=1;

end;

else do;

strata=2; event=0;

end;

run;

** Dataset name: “coloncancer_other”, which is a duplicate of the dataset “coloncancer_dataset”;

** Create a variable named: “strata”, where strata=3.

** Create a variable named: “event”, 1=other treatment’ or 0=did not have other treatment;

data coloncancer_other;

set coloncancer_dataset;

if colontrt=3 then do;

strata=3; event=1;

end;

else do;

strata=3; event=0;

end;

run;

** Dataset name: “coloncancer_died”, which is a duplicate of the dataset “coloncancer_dataset”;

** Create a variable named: “strata”, where strata=4.

** Create a variable named: “event”, 1=assigned to “no treatment, died” or 0=NOT assigned to “no treatment, died”;

data coloncancer_died;

set coloncancer_dataset;

if colontrt=4 then do;

strata=4; event=1;

end;

else do;

strata=4; event=0;

end;

run;

** Append the four datasets;

data colon_jointcox;

set

coloncancer_surgery

coloncancer_surgchemo

coloncancer_other

coloncancer_died

;

run;

Step 2: Use the augmented dataset to apply the joint Cox model in *PHREG*:

* Specify the reference category for each characteristic in the class statement;

* Add the variable “strata” to the class statement;

* Add all characteristics in the model statement;

* Add an interaction term (i.e, *) between the variable “strata” and each characteristic in the model;

* Outcome is the variable “event”, censoring those with no event (i.e, event= 0);

* Add the variable “strata” to the strata statement;

* To account for duplicate records of cases in the dataset, add the unique identifier, named here as “ppn” to the id statement;

* To obtain hazard ratios for variable ”strata” 4=no treatment, died, the hazardratio statement needs to be specified for each characteristic in the model;

proc phreg covs(aggregate) data=colon_jointcox;

class agediagnosis(ref=’45-74’) sex(ref=’Male’) spreaddisease(ref=’Localised’) charlson(ref=’0’) aria(ref=’Major Cities’) insurance(ref=’Yes’) qualification(ref=’University degree or higher’) seifa(ref=’Quintile 1 or 2 (least)’) bmi(ref=’Underweight/Normal’) smoking(ref=’Never’) language(ref=’No’) married(ref=’Yes’) mospf10(ref=’90-100 high function’) emergency(ref=’No’) fobt(ref=’Yes’) sigmoidcolonoscopy(ref=’Yes’) strata /order=internal;

model timetofirsttrt*event(0)= agediagnosis sex spreaddisease charlson aria insurance qualification seifa bmi smoking language married mospf10 emergency fobt sigmoidcolonoscopy

agediagnosis*strata sex*strata spreaddisease*strata charlson*strata aria*strata insurance*strata qualification*strata seifa*strata bmi*strata smoking*strata language*strata married*strata mospf10*strata emergency*strata fobt*strata sigmoidcolonoscopy*strata

/rl;

strata strata;

id ppn;

hazardratio agediagnosis /diff=ref at (strata=’1’);

hazardratio sex /diff=ref at (strata=1);

hazardratio spreaddisease /diff=ref at (strata=’1’);

hazardratio charlson /diff=ref at (strata=’1’);

hazardratio aria /diff=ref at (strata=’1’);

hazardratio insurance /diff=ref at (strata=’1’);

hazardratio qualification /diff=ref at (strata=’1’);

hazardratio seifa /diff=ref at (strata=’1’);

hazardratio bmi /diff=ref at (strata=’1’);

hazardratio smoking /diff=ref at (strata=’1’);

hazardratio language /diff=ref at (strata=’1’);

hazardratio married /diff=ref at (strata=’1’);

hazardratio mospf10 /diff=ref at (strata=’1’);

hazardratio emergency /diff=ref at (strata=’1’);

hazardratio fobt /diff=ref at (strata=’1’);

hazardratio sigmoidcolonoscopy /diff=ref at (strata=’1’);

ods output modelanova=pvalue;

run;

* The p-value for the interaction term between each characteristic and strata represents the overall significance of the characteristic across all treatment outcomes;

**References**

1. Fine JP, Gray RJ: **A Proportional Hazards Model for the Subdistribution of a Competing Risk**. *Journal of the American Statistical Association* 1999, **94**(446):496-509.

2. Lau B, Cole SR, Gange SJ: **Competing Risk Regression Models for Epidemiologic Data**. *American Journal of Epidemiology* 2009, **170**(2):244-256.

3. Putter H, Fiocco M, Geskus RB: **Tutorial in biostatistics: competing risks and multi-state models**. *Stat Med* 2007, **26**(11):2389-2430.

4. Lunn M, McNeil D: **Applying Cox regression to competing risks**. *Biometrics* 1995, **51**(2):524-532.

5. Austin PC, Lee DS, Fine JP: **Introduction to the Analysis of Survival Data in the Presence of Competing Risks**. *Circulation* 2016, **133**(6):601-609.

6. **Using the PHREG procedure to analyze competing risks data** [<https://support.sas.com/rnd/app/stat/papers/2014/competingrisk2014.pdf>]

7. **stcrreg - competing risks regression manual** [<https://www.stata.com/manuals/ststcrreg.pdf>]
