# Supplementary material for: Colon and rectal cancer treatment patterns and their associations with clinical, sociodemographic and lifestyle characteristics: analysis of the Australian 45 and Up Study cohort
Source: BMC Cancer. 2023 Jan 18;23:60. doi: 10.1186/s12885-023-10528-8 (PMC9845101; doi:10.1186/s12885-023-10528-8)
Supplement: Supplementary file 6 — Additional file 6. Selection of colon and rectal cancer cases from the 45 and Up Study cohort for inclusion in this study. [file 12885_2023_10528_MOESM6_ESM.docx]

**
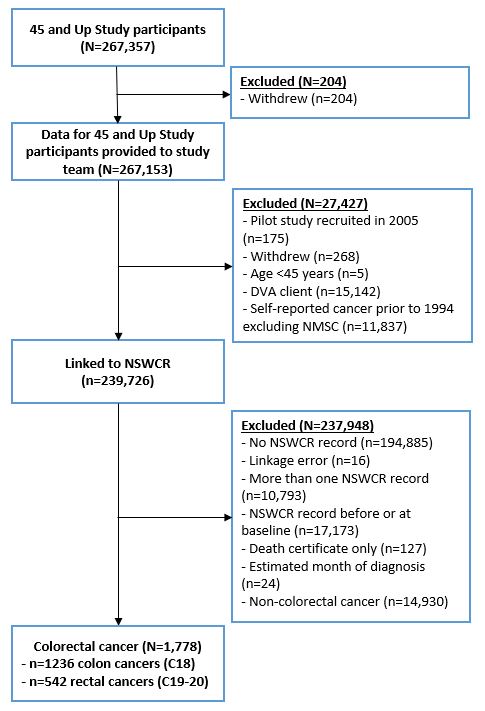
**

**Additional file 6: Selection of colon and rectal cancer cases from the 45 and Up Study cohort for inclusion in this study.**

**DVA: Department of Veterans' Affairs; NMSC: Non-melanoma skin cancers; NSWCR: New South Wales Cancer Registry**
