# Supplementary material for: Colon and rectal cancer treatment patterns and their associations with clinical, sociodemographic and lifestyle characteristics: analysis of the Australian 45 and Up Study cohort
Source: BMC Cancer. 2023 Jan 18;23:60. doi: 10.1186/s12885-023-10528-8 (PMC9845101; doi:10.1186/s12885-023-10528-8)
Supplement: Supplementary file 7 — Additional file 7. Age and spread of disease at diagnosis of colon and rectal cancer cases in the 45 and Up Study, and of all cases in NSW and Australia. [file 12885_2023_10528_MOESM7_ESM.docx]

**Additional file 7. Age and spread of disease at diagnosis of colon and rectal cancer cases in the 45 and Up Study, and of all cases in NSW and Australia**

| **Characteristic** | **Colon cancer cases** | | | **Rectal cancer cases** | | |
| --- | --- | --- | --- | --- | --- | --- |
|  | **45 and Up Study** | **All NSW^1,2^** | **All Australia^3^** | **45 and Up Study** | **All NSW^1,2^** | **All Australia^3^** |
| Age at diagnosis, median | 72 | 72 | 72.1 | 68 | 67 | 67.1 |
| Spread of disease % |  |  |  |  |  |  |
| Localised | 33.4 | 31.6 | − | 33.2 | 36.2 | − |
| Regional | 40.6 | 42.8 | − | 42.1 | 32.5 | − |
| Distant | 20.4 | 18 | − | 16.4 | 15.7 | − |
| Unknown | 5.6 | 7.7 |  | 8.3 | 15.6 |  |

1 Median age at cancer diagnosis for all of NSW obtained from Cancer Incidence and Mortality Report 2010: <https://www.cancer.nsw.gov.au/about-cancer/document-library/cancer-incidence-and-mortality-report-2010>

2 Spread of disease at cancer diagnosis for all of NSW obtained from: <https://www.cancer.nsw.gov.au/research-and-data/cancer-data-and-statistics/cancer-statistics-nsw#//analysis/incidence/>

3 Median age at cancer diagnosis for all of Australia obtained from: <https://www.aihw.gov.au/reports/cancer/cancer-data-in-australia/contents/cancer-incidence-by-age-visualisation>
