# Supplementary material for: Colon and rectal cancer treatment patterns and their associations with clinical, sociodemographic and lifestyle characteristics: analysis of the Australian 45 and Up Study cohort
Source: BMC Cancer. 2023 Jan 18;23:60. doi: 10.1186/s12885-023-10528-8 (PMC9845101; doi:10.1186/s12885-023-10528-8)
Supplement: Supplementary file 8 — Additional file 8. Overall 1-, 2-, 3- and 5-year survival after a diagnosis of colon or rectal cancer, by spread of disease, based on RBDM data to June 2017. [file 12885_2023_10528_MOESM8_ESM.docx]

**Additional file 8. Overall 1-, 2-, 3- and 5-year survival after a diagnosis of colon or rectal cancer, by spread of disease, based on RBDM data to June 2017**

| **Cancer type** | **Overall survival^1^ % (95% CI)** | | | |
| --- | --- | --- | --- | --- |
|  | **1-year** | **2-years** | **3-years** | **5-years** |
| **Colon cancer (N=1236)** |  |  |  |  |
| All | 84.2 (82.1-86.2) | 76.4 (74.0-78.7) | 71.7 (69.2-74.2) | 63.7 (60.9-66.5) |
| Localised | 96.4 (94.2-97.9) | 92.7 (90.0-95.0) | 89.8 (86.7-92.5) | 85.3 (81.6-88.7) |
| Regional | 91.8 (89.2-94.0) | 84.5 (81.2-87.5) | 80.3 (76.7-83.6) | 68.6 (64.3-72.8) |
| Distant | 49.6 (43.6-55.9) | 31.7 (26.4-37.9) | 23.0 (18.3-28.7) | 15.9 (11.9-21.1) |
| Unknown | 82.6 (72.8-90.4) | 82.6 (72.8-90.4) | 78.3 (68.0-87.1) | 74.5 (63.5-84.3) |
| **Rectal cancer (N=542)** |  |  |  |  |
| All | 87.1 (84.1-89.7) | 81.2 (77.8-84.3) | 75.3 (71.6-78.8) | 66.7 (62.6-70.7) |
| Localised | 96.1 (92.5-98.3) | 93.9 (89.7-96.8) | 88.9 (83.8-92.9) | 84.0 (78.1-89.0) |
| Regional | 89.9 (85.6-93.4) | 86.4 (81.6-90.5) | 79.8 (74.4-84.8) | 71.0 (64.9-76.9) |
| Distant | 61.8 (51.9-71.8) | 40.4 (31.1-51.4) | 31.5 (22.9-42.2) | 15.7 (9.5-25.4) |
| Unknown | 86.7 (75.1-94.6) | 84.4 (72.5-93.2) | 84.4 (72.5-93.2) | 77.4 (64.3-88.3) |

1 Overall survival treats all deaths as the event, censoring those alive at the end of follow-up
