# Supplementary material for: Colon and rectal cancer treatment patterns and their associations with clinical, sociodemographic and lifestyle characteristics: analysis of the Australian 45 and Up Study cohort
Source: BMC Cancer. 2023 Jan 18;23:60. doi: 10.1186/s12885-023-10528-8 (PMC9845101; doi:10.1186/s12885-023-10528-8)
Supplement: Supplementary file 9 — Additional file 9. Cancer-specific 1- and 2-year survival after a diagnosis of colon or rectal cancer, by spread of disease, based on NSWCR data to 31 December 2013. [file 12885_2023_10528_MOESM9_ESM.docx]

**Additional file 9. Cancer-specific 1- and 2-year survival after a diagnosis of colon or rectal cancer, by spread of disease, based on NSWCR data to 31 December 2013**

| **Cancer type** | **Cancer-specific survival % (95% CI)** | |
| --- | --- | --- |
|  | **1-year** | **2-years** |
| **Colon cancer^1^ (N=1236)** |  |  |
| All | 86.4 (84.3-88.3) | 79.8 (77.3-82.2) |
| Localised | 98.4 (96.8-99.3) | 97.5 (95.4-98.8) |
| Regional | 93.9 (91.5-95.9) | 88.1 (84.6-91.1) |
| Distant | 52.7 (46.6-59.2) | 35.9 (30.1-42.5) |
| Unknown | 86.6 (76.6-93.8) | 86.6 (76.6-93.8) |
| **Rectal cancer^2^ (N=542)** |  |  |
| All | 90.4 (87.6-92.8) | 84.3 (80.8-87.5) |
| Localised | 96.9 (93.3-98.8) | 95.5 (91.3-98.0) |
| Regional | 94.7 (91.0-97.2) | 91.1 (86.5-94.6) |
| Distant | 65.5 (55.2-75.5) | 43.3 (33.1-55.2) |
| Unknown | 93.2 (83.2-98.2) | 90.5 (79.4-97.0) |

1 Colon cancer-specific survival treats death from colon cancer (C18) as the event of interest, death from other causes as the competing event, censoring those alive at the end of follow-up

2 Rectal cancer-specific survival treats death from rectal cancer (C19-20) as the event of interest, death from other causes as the competing event, censoring those alive at the end of follow-up
