# Supplementary material for: Colon and rectal cancer treatment patterns and their associations with clinical, sociodemographic and lifestyle characteristics: analysis of the Australian 45 and Up Study cohort
Source: BMC Cancer. 2023 Jan 18;23:60. doi: 10.1186/s12885-023-10528-8 (PMC9845101; doi:10.1186/s12885-023-10528-8)
Supplement: Supplementary file 10 — Additional file 10. Time from diagnosis to first cancer treatment received in the 0-2 years after diagnosis, by treatment category. The cumulative percentage was obtained using the cumulative incidence function (CIF). [file 12885_2023_10528_MOESM10_ESM.docx]

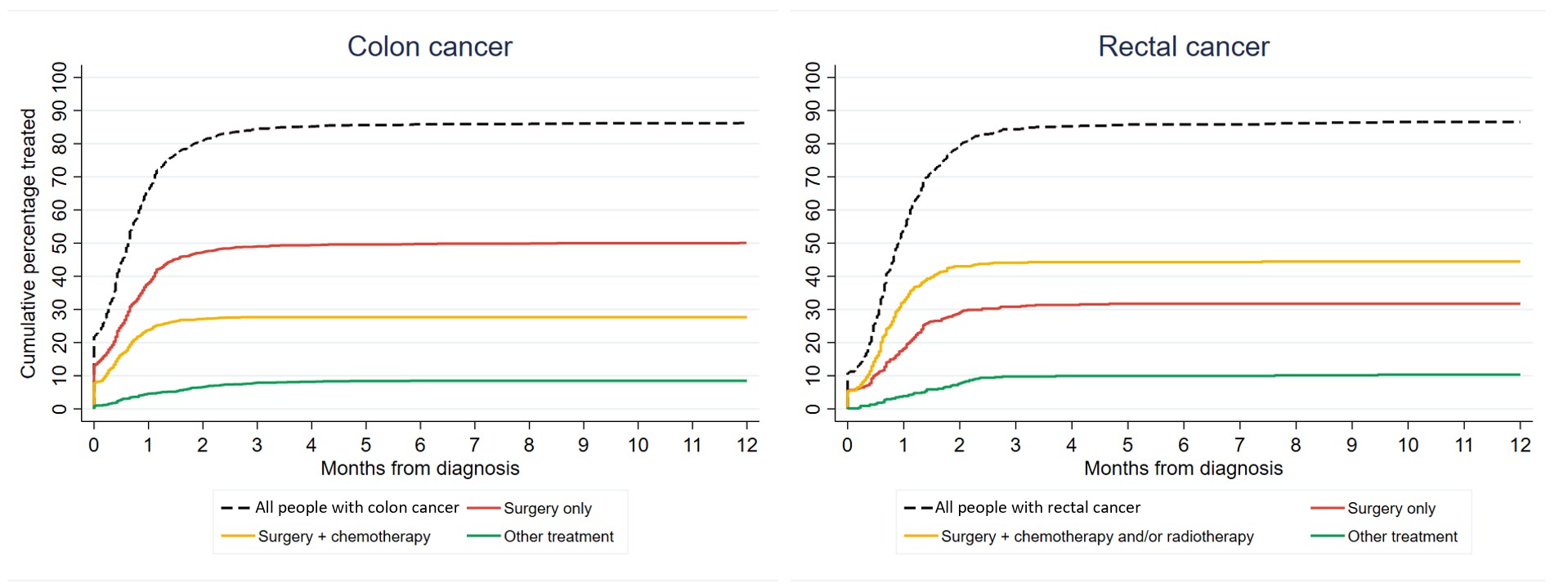


**Additional file 10: Time from diagnosis to first cancer treatment received in the 0-2 years after diagnosis, by treatment category. The cumulative percentage was obtained using the cumulative incidence function (CIF).**
