# Supplementary material for: Colon and rectal cancer treatment patterns and their associations with clinical, sociodemographic and lifestyle characteristics: analysis of the Australian 45 and Up Study cohort
Source: BMC Cancer. 2023 Jan 18;23:60. doi: 10.1186/s12885-023-10528-8 (PMC9845101; doi:10.1186/s12885-023-10528-8)
Supplement: Supplementary file 12 — Additional file 12. Multivariable adjusted hazard ratios for associations between characteristics of colon cancer cases and cancer treatment received within 2 years after cancer diagnosis. Subdistribution hazard ratios (SHRs) from the competing risks Fine-Gray model, and hazard ratios (HRs) from the cause-specific Cox hazard and joint Cox model were adjusted for all characteristics shown in the table. P-values with two asterisks (**) are significant after Bonferroni adjustment for 16 tests (i.e., p<0.003), while those with one asterisk (*) are significant at a nominal level of p<0.05. [file 12885_2023_10528_MOESM12_ESM.docx]

**Additional file 12. Multivariable adjusted hazard ratios for associations between characteristics of colon cancer cases and cancer treatment received within 2 years after cancer diagnosis. Subdistribution hazard ratios (SHRs) from the competing risks Fine-Gray model, and hazard ratios (HRs) from the cause-specific Cox hazard and joint Cox model were adjusted for all characteristics shown in the table. P-values with two asterisks (**) are significant after Bonferroni adjustment for 16 tests (i.e., p<0.003), while those with one asterisk (*) are significant at a nominal level of p<0.05.**

| **Characteristics** | **No treatment (died)** | |  | **Surgery Only** |  |  | **Surgery plus chemotherapy** | |  | **Other treatment** |  |  | **Joint Cox Model overall p-value** |
| --- | --- | --- | --- | --- | --- | --- | --- | --- | --- | --- | --- | --- | --- |
|  | **Fine-Gray Model** | **Cox Model** | **Joint Cox Model** | **Fine-Gray Model** | **Cox Model** | **Joint Cox Model** | **Fine-Gray Model** | **Cox Model** | **Joint Cox Model** | **Fine-Gray Model** | **Cox Model** | **Joint Cox Model** |  |
|  | **SHR (95% CI)** | **HR (95% CI)** | **HR (95% CI)** | **SHR (95% CI)** | **HR (95% CI)** | **HR (95% CI)** | **SHR (95% CI)** | **HR (95% CI)** | **HR (95% CI)** | **SHR (95% CI)** | **HR (95% CI)** | **HR (95% CI)** |  |
| **Age at diagnosis (years)** |  |  |  |  |  |  |  |  |  |  |  |  |  |
| 45-74 | 1.0 (Ref) | 1.0 (Ref) | 1.0 (Ref) | 1.0 (Ref) | 1.0 (Ref) | 1.0 (Ref) | 1.0 (Ref) | 1.0 (Ref) | 1.0 (Ref) | 1.0 (Ref) | 1.0 (Ref) | 1.0 (Ref) |  |
| ≥75 | 3.6 (1.8-7.1) | 2.4 (1.3-4.3) | 2.4 (1.3-4.2) | 1.5 (1.3-1.8) | 1.3 (1.1-1.6) | 1.3 (1.1-1.6) | 0.4 (0.3-0.6) | 0.5 (0.4-0.6) | 0.5 (0.4-0.6) | 0.6 (0.3-1.0) | 0.5 (0.3-0.8) | 0.5 (0.3-0.9) |  |
| *p-value* | *<0.001*** | *0.005** | *0.003*** | *<0.001*** | *0.003*** | *0.002*** | *<0.001*** | *<0.001*** | *<0.001*** | *0.032** | *0.008** | *0.012** | *<0.001*** |
| **Sex** |  |  |  |  |  |  |  |  |  |  |  |  |  |
| Male | 1.0 (Ref) | 1.0 (Ref) | 1.0 (Ref) | 1.0 (Ref) | 1.0 (Ref) | 1.0 (Ref) | 1.0 (Ref) | 1.0 (Ref) | 1.0 (Ref) | 1.0 (Ref) | 1.0 (Ref) | 1.0 (Ref) |  |
| Female | 0.6 (0.3-1.0) | 0.7 (0.4-1.3) | 0.7 (0.4-1.3) | 1.0 (0.9-1.2) | 1.0 (0.9-1.2) | 1.0 (0.9-1.2) | 1.1 (0.9-1.4) | 1.1 (0.9-1.4) | 1.1 (0.9-1.4) | 1.0 (0.6-1.5) | 1.2 (0.8-1.8) | 1.2 (0.7-1.9) |  |
| *p-value* | *0.042** | *0.266* | *0.281* | *0.785* | *0.629* | *0.615* | *0.248* | *0.304* | *0.283* | *0.956* | *0.479* | *0.491* | *0.569* |
| **Spread of disease** |  |  |  |  |  |  |  |  |  |  |  |  |  |
| Localised | 1.0 (Ref) | 1.0 (Ref) | 1.0 (Ref) | 1.0 (Ref) | 1.0 (Ref) | 1.0 (Ref) | 1.0 (Ref) | 1.0 (Ref) | 1.0 (Ref) | 1.0 (Ref) | 1.0 (Ref) | 1.0 (Ref) |  |
| Regional | 1.0 (0.3-3.0) | 2.9 (0.9-9.7) | 2.9 (0.9-9.3) | 0.4 (0.3-0.5) | 0.7 (0.6-0.8) | 0.7 (0.6-0.8) | 11.6 (7.4-18.3) | 11.1 (7.0-17.4) | 11.1 (7.0-17.5) | 3.5 (1.6-7.6) | 5.6 (2.5-12.3) | 5.6 (2.5-12.5) |  |
| Distant | 13.6 (5.5-33.9) | 52.1 (16.7-162.6) | 52.1 (16.2-167.0) | 0.1 (0.1-0.2) | 0.2 (0.2-0.3) | 0.2 (0.2-0.3) | 9.0 (5.5-14.7) | 7.4 (4.6-12.1) | 7.4 (4.5-12.2) | 15.1 (6.8-33.2) | 16.9 (7.8-36.5) | 16.9 (7.6-37.7) |  |
| Unknown | 10.3 (3.3-31.9) | 5.1 (1.4-18.8) | 5.1 (1.1-22.7) | 0.4 (0.3-0.6) | 0.4 (0.3-0.6) | 0.4 (0.3-0.6) | 2.3 (1.0-5.2) | 1.6 (0.7-3.6) | 1.6 (0.7-3.7) | 2.8 (0.8-9.7) | 1.4 (0.4-4.9) | 1.4 (0.4-5.3) |  |
| *p-value* | *<0.001*** | *<0.001*** | *<0.001*** | *<0.001*** | *<0.001*** | *<0.001*** | *<0.001*** | *<0.001*** | *<0.001*** | *<0.001*** | *<0.001*** | *<0.001*** | *<0.001*** |
| **Charlsons comorbidity index** |  |  |  |  |  |  |  |  |  |  |  |  |  |
| 0 | 1.0 (Ref) | 1.0 (Ref) | 1.0 (Ref) | 1.0 (Ref) | 1.0 (Ref) | 1.0 (Ref) | 1.0 (Ref) | 1.0 (Ref) | 1.0 (Ref) | 1.0 (Ref) | 1.0 (Ref) | 1.0 (Ref) |  |
| 1 | 2.0 (0.7-5.5) | 2.9 (1.1-7.9) | 2.9 (1.3-6.9) | 1.2 (0.9-1.7) | 1.2 (0.8-1.6) | 1.2 (0.8-1.6) | 0.5 (0.3-1.0) | 0.5 (0.3-1.1) | 0.5 (0.3-1.0) | 1.2 (0.5-3.1) | 0.9 (0.4-2.4) | 0.9 (0.4-2.2) |  |
| ≥2 | 1.6 (0.7-3.6) | 2.7 (1.2-6.1) | 2.7 (1.1-6.3) | 1.2 (0.8-1.7) | 1.1 (0.8-1.6) | 1.1 (0.7-1.6) | 0.5 (0.2-1.1) | 0.5 (0.2-1.1) | 0.5 (0.2-1.1) | 2.2 (0.9-5.3) | 1.7 (0.7-3.8) | 1.7 (0.7-3.9) |  |
| *p-value* | *0.237* | *0.015** | *0.010** | *0.440* | *0.672* | *0.656* | *0.043** | *0.051** | *0.046** | *0.186* | *0.444* | *0.467* | *0.009** |
| **MOSPF-10 physical functioning scale** |  |  |  |  |  |  |  |  |  |  |  |  |  |
| 90-100 (high function) | 1.0 (Ref) | 1.0 (Ref) | 1.0 (Ref) | 1.0 (Ref) | 1.0 (Ref) | 1.0 (Ref) | 1.0 (Ref) | 1.0 (Ref) | 1.0 (Ref) | 1.0 (Ref) | 1.0 (Ref) | 1.0 (Ref) |  |
| 60-89 | 1.2 (0.6-2.6) | 1.2 (0.6-2.5) | 1.2 (0.6-2.5) | 1.0 (0.8-1.2) | 1.0 (0.8-1.3) | 1.0 (0.8-1.3) | 1.2 (0.9-1.5) | 1.2 (0.9-1.6) | 1.2 (0.9-1.5) | 0.7 (0.4-1.1) | 0.7 (0.4-1.1) | 0.7 (0.4-1.1) |  |
| 0-59 (low function) | 2.4 (1.2-4.9) | 3.5 (1.6-7.4) | 3.5 (1.6-7.6) | 1.1 (0.9-1.4) | 1.2 (0.9-1.5) | 1.2 (0.9-1.5) | 0.8 (0.6-1.3) | 1.0 (0.7-1.5) | 1.0 (0.6-1.5) | 0.6 (0.3-1.1) | 1.0 (0.5-1.9) | 1.0 (0.5-1.8) |  |
| Unspecified | 2.0 (0.8-5.1) | 2.3 (1.0-5.7) | 2.3 (0.9-6.0) | 0.8 (0.6-1.0) | 0.9 (0.6-1.2) | 0.9 (0.7-1.2) | 1.7 (1.2-2.4) | 1.6 (1.1-2.3) | 1.6 (1.1-2.2) | 0.5 (0.2-1.2) | 0.5 (0.2-1.2) | 0.5 (0.2-1.2) |  |
| *p-value* | *0.087* | *0.005** | *0.014** | *0.152* | *0.359* | *0.360* | *0.008** | *0.087* | *0.061* | *0.142* | *0.242* | *0.224* | *0.012** |
| **Emergency presentation prior to diagnosis** |  |  |  |  |  |  |  |  |  |  |  |  |  |
| Yes | 2.9 (1.6-5.2) | 1.9 (1.1-3.5) | 1.9 (1.1-3.4) | 0.8 (0.6-1.0) | 0.9 (0.7-1.1) | 0.9 (0.7-1.2) | 1.2 (0.9-1.6) | 1.2 (0.9-1.6) | 1.2 (0.9-1.6) | 0.9 (0.5-1.4) | 0.8 (0.5-1.3) | 0.8 (0.5-1.3) |  |
| No | 1.0 (Ref) | 1.0 (Ref) | 1.0 (Ref) | 1.0 (Ref) | 1.0 (Ref) | 1.0 (Ref) | 1.0 (Ref) | 1.0 (Ref) | 1.0 (Ref) | 1.0 (Ref) | 1.0 (Ref) | 1.0 (Ref) |  |
| *p-value* | *<0.001*** | *0.029** | *0.027** | *0.098* | *0.359* | *0.387* | *0.159* | *0.156* | *0.169* | *0.557* | *0.389* | *0.403* | *0.060* |
| **Smoking status** |  |  |  |  |  |  |  |  |  |  |  |  |  |
| Never smoker | 1.0 (Ref) | 1.0 (Ref) | 1.0 (Ref) | 1.0 (Ref) | 1.0 (Ref) | 1.0 (Ref) | 1.0 (Ref) | 1.0 (Ref) | 1.0 (Ref) | 1.0 (Ref) | 1.0 (Ref) | 1.0 (Ref) |  |
| Former smoker (quit > 15 years) | 1.4 (0.8-2.5) | 1.6 (0.8-3.1) | 1.6 (0.8-2.9) | 0.9 (0.8-1.1) | 0.9 (0.7-1.1) | 0.9 (0.8-1.1) | 1.0 (0.8-1.3) | 0.9 (0.7-1.2) | 0.9 (0.7-1.2) | 0.9 (0.5-1.5) | 1.0 (0.6-1.6) | 1.0 (0.6-1.6) |  |
| Current/Former smoker (quit ≤ 15 years) | 1.1 (0.5-2.5) | 1.1 (0.5-2.1) | 1.1 (0.5-2.2) | 0.9 (0.7-1.1) | 0.8 (0.7-1.1) | 0.8 (0.7-1.1) | 1.2 (0.9-1.5) | 1.0 (0.8-1.4) | 1.0 (0.8-1.4) | 0.8 (0.5-1.3) | 0.8 (0.5-1.4) | 0.8 (0.5-1.3) |  |
| *p-value* | *0.565* | *0.432* | *0.362* | *0.387* | *0.323* | *0.299* | *0.487* | *0.822* | *0.802* | *0.635* | *0.701* | *0.663* | *0.624* |
| **Body Mass Index (kg/m^2^)** |  |  |  |  |  |  |  |  |  |  |  |  |  |
| Underweight/Normal (<25) | 1.0 (Ref) | 1.0 (Ref) | 1.0 (Ref) | 1.0 (Ref) | 1.0 (Ref) | 1.0 (Ref) | 1.0 (Ref) | 1.0 (Ref) | 1.0 (Ref) | 1.0 (Ref) | 1.0 (Ref) | 1.0 (Ref) |  |
| Overweight/Obese (≥25) | 0.7 (0.4-1.3) | 0.5 (0.3-1.0) | 0.5 (0.3-1.0) | 1.0 (0.9-1.2) | 1.1 (0.9-1.3) | 1.1 (0.9-1.3) | 1.0 (0.7-1.2) | 0.9 (0.7-1.2) | 0.9 (0.7-1.2) | 1.4 (0.9-2.3) | 1.5 (0.9-2.4) | 1.5 (0.9-2.4) |  |
| Unspecified | 1.0 (0.4-2.5) | 0.5 (0.2-1.3) | 0.5 (0.2-1.2) | 0.8 (0.5-1.1) | 0.8 (0.6-1.2) | 0.8 (0.6-1.2) | 0.9 (0.6-1.5) | 1.0 (0.6-1.5) | 1.0 (0.6-1.5) | 2.3 (1.1-4.8) | 2.1 (0.9-4.7) | 2.1 (1.0-4.7) |  |
| *p-value* | *0.347* | *0.081* | *0.122* | *0.288* | *0.333* | *0.321* | *0.926* | *0.858* | *0.859* | *0.076* | *0.137* | *0.139* | *0.131* |
| **Ever had FOBT** |  |  |  |  |  |  |  |  |  |  |  |  |  |
| Yes | 1.0 (Ref) | 1.0 (Ref) | 1.0 (Ref) | 1.0 (Ref) | 1.0 (Ref) | 1.0 (Ref) | 1.0 (Ref) | 1.0 (Ref) | 1.0 (Ref) | 1.0 (Ref) | 1.0 (Ref) | 1.0 (Ref) |  |
| No | 1.2 (0.6-2.3) | 0.5 (0.2-1.1) | 0.5 (0.2-1.1) | 1.1 (0.9-1.3) | 1.1 (0.9-1.3) | 1.1 (0.9-1.3) | 0.9 (0.7-1.2) | 0.9 (0.7-1.2) | 0.9 (0.7-1.2) | 0.8 (0.5-1.3) | 0.5 (0.3-0.9) | 0.5 (0.4-0.9) |  |
| *p-value* | *0.678* | *0.071* | *0.068* | *0.349* | *0.572* | *0.526* | *0.534* | *0.500* | *0.472* | *0.318* | *0.018** | *0.007** | *0.014** |
| **Ever had sigmoidoscopy/colonoscopy** | |  |  |  |  |  |  |  |  |  |  |  |  |
| Yes | 1.0 (Ref) | 1.0 (Ref) | 1.0 (Ref) | 1.0 (Ref) | 1.0 (Ref) | 1.0 (Ref) | 1.0 (Ref) | 1.0 (Ref) | 1.0 (Ref) | 1.0 (Ref) | 1.0 (Ref) | 1.0 (Ref) |  |
| No | 1.1 (0.6-2.0) | 1.7 (0.9-3.2) | 1.7 (0.9-3.3) | 1.0 (0.8-1.1) | 1.0 (0.8-1.2) | 1.0 (0.8-1.2) | 1.2 (0.9-1.5) | 1.2 (0.9-1.5) | 1.2 (0.9-1.5) | 0.8 (0.5-1.4) | 1.0 (0.6-1.6) | 1.0 (0.6-1.6) |  |
| *p-value* | *0.803* | *0.100* | *0.128* | *0.659* | *0.849* | *0.844* | *0.186* | *0.190* | *0.168* | *0.478* | *0.976* | *0.975* | *0.360* |
| **Place of residence** |  |  |  |  |  |  |  |  |  |  |  |  |  |
| Major City | 1.0 (Ref) | 1.0 (Ref) | 1.0 (Ref) | 1.0 (Ref) | 1.0 (Ref) | 1.0 (Ref) | 1.0 (Ref) | 1.0 (Ref) | 1.0 (Ref) | 1.0 (Ref) | 1.0 (Ref) | 1.0 (Ref) |  |
| Other | 0.6 (0.4-1.2) | 0.7 (0.4-1.4) | 0.7 (0.4-1.5) | 1.2 (1.0-1.5) | 1.3 (1.0-1.5) | 1.3 (1.0-1.5) | 0.9 (0.7-1.1) | 0.9 (0.7-1.2) | 0.9 (0.7-1.2) | 1.3 (0.9-2.1) | 1.6 (1.0-2.7) | 1.6 (1.1-2.6) |  |
| *p-value* | *0.151* | *0.356* | *0.397* | *0.037** | *0.027** | *0.025** | *0.266* | *0.617* | *0.612* | *0.197* | *0.047** | *0.030** | *0.087* |
| **Area-level SES** |  |  |  |  |  |  |  |  |  |  |  |  |  |
| Quintile 4 or 5 (least disadvantaged) | 1.0 (Ref) | 1.0 (Ref) | 1.0 (Ref) | 1.0 (Ref) | 1.0 (Ref) | 1.0 (Ref) | 1.0 (Ref) | 1.0 (Ref) | 1.0 (Ref) | 1.0 (Ref) | 1.0 (Ref) | 1.0 (Ref) |  |
| Quintile 2 or 3 | 1.2 (0.6-2.3) | 1.1 (0.5-2.3) | 1.1 (0.5-2.3) | 0.9 (0.8-1.1) | 0.9 (0.8-1.2) | 0.9 (0.8-1.2) | 0.8 (0.6-1.1) | 0.9 (0.7-1.2) | 0.9 (0.7-1.2) | 1.3 (0.8-2.1) | 1.1 (0.6-1.9) | 1.1 (0.6-1.8) |  |
| Quintile 1 (most disadvantaged) | 1.7 (0.8-3.8) | 1.6 (0.7-3.7) | 1.6 (0.6-3.8) | 0.7 (0.6-1.0) | 0.7 (0.5-1.0) | 0.7 (0.5-1.0) | 1.1 (0.8-1.6) | 1.1 (0.8-1.5) | 1.1 (0.8-1.5) | 1.0 (0.5-1.9) | 0.8 (0.4-1.7) | 0.8 (0.4-1.7) |  |
| *p-value* | *0.388* | *0.509* | *0.526* | *0.108* | *0.067* | *0.064* | *0.091* | *0.474* | *0.436* | *0.501* | *0.682* | *0.698* | *0.246* |
| **Highest qualification** |  |  |  |  |  |  |  |  |  |  |  |  |  |
| University degree or higher | 1.0 (Ref) | 1.0 (Ref) | 1.0 (Ref) | 1.0 (Ref) | 1.0 (Ref) | 1.0 (Ref) | 1.0 (Ref) | 1.0 (Ref) | 1.0 (Ref) | 1.0 (Ref) | 1.0 (Ref) | 1.0 (Ref) |  |
| School/Higher school/Trade/Certificate | 2.0 (0.7-5.4) | 1.8 (0.6-5.5) | 1.8 (0.7-4.4) | 0.9 (0.8-1.2) | 0.9 (0.7-1.1) | 0.9 (0.7-1.1) | 0.9 (0.7-1.2) | 0.9 (0.7-1.2) | 0.9 (0.7-1.1) | 1.3 (0.7-2.3) | 1.0 (0.6-1.9) | 1.0 (0.6-1.9) |  |
| No school certificate or qualification | 1.8 (0.6-5.5) | 1.6 (0.5-5.5) | 1.6 (0.6-4.2) | 1.1 (0.8-1.5) | 1.0 (0.7-1.3) | 1.0 (0.7-1.3) | 0.6 (0.4-1.0) | 0.6 (0.4-1.0) | 0.6 (0.4-1.0) | 1.2 (0.6-2.7) | 1.1 (0.5-2.4) | 1.1 (0.5-2.3) |  |
| *p-value* | *0.385* | *0.587* | *0.442* | *0.337* | *0.419* | *0.372* | *0.105* | *0.123* | *0.125* | *0.736* | *0.982* | *0.978* | *0.414* |
| **Private health insurance** |  |  |  |  |  |  |  |  |  |  |  |  |  |
| Yes | 1.0 (Ref) | 1.0 (Ref) | 1.0 (Ref) | 1.0 (Ref) | 1.0 (Ref) | 1.0 (Ref) | 1.0 (Ref) | 1.0 (Ref) | 1.0 (Ref) | 1.0 (Ref) | 1.0 (Ref) | 1.0 (Ref) |  |
| No | 1.1 (0.6-2.0) | 1.2 (0.6-2.1) | 1.2 (0.6-2.3) | 0.9 (0.8-1.1) | 0.8 (0.6-0.9) | 0.8 (0.6-0.9) | 0.7 (0.6-0.9) | 0.6 (0.5-0.8) | 0.6 (0.5-0.8) | 1.2 (0.8-1.9) | 0.8 (0.5-1.3) | 0.8 (0.5-1.2) |  |
| *p-value* | *0.644* | *0.625* | *0.656* | *0.228* | *0.003*** | *0.003*** | *0.017** | *<0.001*** | *<0.001*** | *0.423* | *0.322* | *0.305* | *0.296* |
| **Married or de-facto** |  |  |  |  |  |  |  |  |  |  |  |  |  |
| Yes | 1.0 (Ref) | 1.0 (Ref) | 1.0 (Ref) | 1.0 (Ref) | 1.0 (Ref) | 1.0 (Ref) | 1.0 (Ref) | 1.0 (Ref) | 1.0 (Ref) | 1.0 (Ref) | 1.0 (Ref) | 1.0 (Ref) |  |
| No | 1.4 (0.8-2.5) | 2.2 (1.2-4.0) | 2.2 (1.2-4.0) | 1.1 (0.9-1.3) | 1.1 (0.9-1.3) | 1.1 (0.9-1.3) | 0.8 (0.6-1.0) | 0.8 (0.6-1.1) | 0.8 (0.6-1.1) | 1.2 (0.8-1.9) | 1.3 (0.8-2.1) | 1.3 (0.8-2.0) |  |
| *p-value* | *0.289* | *0.010** | *0.010** | *0.245* | *0.244* | *0.234* | *0.065* | *0.121* | *0.120* | *0.411* | *0.290* | *0.275* | *0.013** |
| **Language other than English** |  |  |  |  |  |  |  |  |  |  |  |  |  |
| Yes | 1.5 (0.7-3.3) | 2.1 (0.9-4.8) | 2.1 (1.0-4.3) | 1.1 (0.8-1.5) | 1.1 (0.8-1.6) | 1.1 (0.8-1.5) | 0.7 (0.5-1.2) | 0.7 (0.5-1.2) | 0.7 (0.5-1.2) | 1.3 (0.6-2.7) | 1.5 (0.7-3.3) | 1.5 (0.8-3.0) |  |
| No | 1.0 (Ref) | 1.0 (Ref) | 1.0 (Ref) | 1.0 (Ref) | 1.0 (Ref) | 1.0 (Ref) | 1.0 (Ref) | 1.0 (Ref) | 1.0 (Ref) | 1.0 (Ref) | 1.0 (Ref) | 1.0 (Ref) |  |
| *p-value* | *0.272* | *0.070* | *0.038** | *0.611* | *0.493* | *0.476* | *0.187* | *0.204* | *0.202* | *0.559* | *0.282* | *0.219* | *0.076* |
