# Supplementary material for: Colon and rectal cancer treatment patterns and their associations with clinical, sociodemographic and lifestyle characteristics: analysis of the Australian 45 and Up Study cohort
Source: BMC Cancer. 2023 Jan 18;23:60. doi: 10.1186/s12885-023-10528-8 (PMC9845101; doi:10.1186/s12885-023-10528-8)
Supplement: Supplementary file 13 — Additional file 13. Multivariable adjusted hazard ratios for associations between characteristics of rectal cancer cases and treatment received within 2 years from the cancer diagnosis. SHRs from the competing risks Fine-Gray model, and HRs from the cause-specific Cox hazard and joint Cox model were adjusted for all characteristics in the table. P-values with two asterisks (**) are significant after Bonferroni adjustment for 16 tests (i.e., p<0.003), while those with one asterisk (*) are significant at a nominal level of p<0.05. [file 12885_2023_10528_MOESM13_ESM.docx]

**Additional file 13. Multivariable adjusted hazard ratios for associations between characteristics of rectal cancer cases and treatment received within 2 years from the cancer diagnosis. SHRs from the competing risks Fine-Gray model, and HRs from the cause-specific Cox hazard and joint Cox model were adjusted for all characteristics in the table. P-values with two asterisks (**) are significant after Bonferroni adjustment for 16 tests (i.e., p<0.003), while those with one asterisk (*) are significant at a nominal level of p<0.05.**

| **Characteristics** | **No treatment (died)** | |  | **Surgery only** |  |  | **Surgery plus chemotherapy and or radiotherapy** | |  | **Other treatment** |  |  | **Joint Cox Model  p-value** |
| --- | --- | --- | --- | --- | --- | --- | --- | --- | --- | --- | --- | --- | --- |
|  | **Fine-Gray Model** | **Cox Model** | **Joint Cox Model** | **Fine-Gray Model** | **Cox Model** | **Joint Cox Model** | **Fine-Gray Model** | **Cox Model** | **Joint Cox Model** | **Fine-Gray Model** | **Cox Model** | **Joint Cox Model** |  |
|  | **SHR (95% CI)** | **HR (95% CI)** | **HR (95% CI)** | **SHR (95% CI)** | **HR (95% CI)** | **HR (95% CI)** | **SHR (95% CI)** | **HR (95% CI)** | **HR (95% CI)** | **SHR (95% CI)** | **HR (95% CI)** | **HR (95% CI)** |  |
| **Age at diagnosis (years)** |  |  |  |  |  |  |  |  |  |  |  |  |  |
| 45-74 | 1.0 (Ref) | 1.0 (Ref) | 1.0 (Ref) | 1.0 (Ref) | 1.0 (Ref) | 1.0 (Ref) | 1.0 (Ref) | 1.0 (Ref) | 1.0 (Ref) | 1.0 (Ref) | 1.0 (Ref) | 1.0 (Ref) |  |
| ≥75 | 6.6 (1.9-23.0) | 6.1 (1.4-27.1) | 6.1 (1.3-29.0) | 1.5 (1.0-2.2) | 1.4 (1.0-2.0) | 1.4 (1.0-2.0) | 0.6 (0.4-0.8) | 0.6 (0.4-0.9) | 0.6 (0.4-0.9) | 0.8 (0.4-1.8) | 0.7 (0.3-1.4) | 0.7 (0.3-1.4) |  |
| *p-value* | *0.003*** | *0.018** | *0.023*** | *0.035*** | *0.080*** | *0.090*** | *0.002*** | *0.013*** | *0.012*** | *0.661* | *0.271* | *0.278* | *0.003*** |
| **Sex** |  |  |  |  |  |  |  |  |  |  |  |  |  |
| Male | 1.0 (Ref) | 1.0 (Ref) | 1.0 (Ref) | 1.0 (Ref) | 1.0 (Ref) | 1.0 (Ref) | 1.0 (Ref) | 1.0 (Ref) | 1.0 (Ref) | 1.0 (Ref) | 1.0 (Ref) | 1.0 (Ref) |  |
| Female | 0.4 (0.1-1.5) | 0.6 (0.2-2.0) | 0.6 (0.2-2.1) | 1.4 (1.0-2.0) | 1.3 (0.9-1.9) | 1.3 (0.9-1.8) | 0.9 (0.7-1.2) | 0.9 (0.7-1.2) | 0.9 (0.7-1.2) | 2.1 (1.1-4.0) | 2.2 (1.1-4.3) | 2.2 (1.0-4.5) |  |
| *p-value* | *0.160* | *0.403* | *0.407* | *0.035** | *0.121* | *0.111* | *0.559* | *0.523* | *0.506* | *0.023** | *0.030** | *0.039** | *0.071* |
| **Spread of disease** |  |  |  |  |  |  |  |  |  |  |  |  |  |
| Localised | 1.0 (Ref) | 1.0 (Ref) | 1.0 (Ref) | 1.0 (Ref) | 1.0 (Ref) | 1.0 (Ref) | 1.0 (Ref) | 1.0 (Ref) | 1.0 (Ref) | 1.0 (Ref) | 1.0 (Ref) | 1.0 (Ref) |  |
| Regional | 1.5 (0.2-11.1) | 6.8 (0.7-68.0) | 6.8 (0.2-184.2) | 0.3 (0.2-0.4) | 0.5 (0.3-0.7) | 0.5 (0.3-0.7) | 5.2 (3.6-7.7) | 4.5 (3.1-6.5) | 4.5 (3.1-6.6) | 1.8 (0.7-4.7) | 3.1 (1.1-8.4) | 3.1 (1.0-9.4) |  |
| Distant | 19.1 (2.1-177.4) | 272.0 (19.9-3,723.7) | 272.0 (5.7-13,033.4) | 0.1 (0.1-0.3) | 0.2 (0.1-0.5) | 0.2 (0.1-0.5) | 2.7 (1.6-4.4) | 2.6 (1.6-4.2) | 2.6 (1.6-4.2) | 8.4 (3.1-22.4) | 13.6 (5.1-36.0) | 13.6 (4.6-39.7) |  |
| Unknown | 8.9 (1.3-61.6) | 34.3 (3.0-393.1) | 34.3 (1.1-1,054.1) | 0.6 (0.3-1.1) | 0.6 (0.3-1.1) | 0.6 (0.3-1.1) | 0.6 (0.2-1.4) | 0.5 (0.2-1.1) | 0.5 (0.2-1.2) | 1.9 (0.4-7.7) | 1.8 (0.4-7.4) | 1.8 (0.4-8.1) |  |
| *p-value* | *0.002*** | *<0.001*** | *<0.001*** | *<0.001*** | *<0.001*** | *<0.001*** | *<0.001*** | *<0.001*** | *<0.001*** | *<0.001*** | *<0.001*** | *<0.001*** | *<0.001*** |
| **Charlsons comorbidity index** |  |  |  |  |  |  |  |  |  |  |  |  |  |
| 0 | 1.0 (Ref) | 1.0 (Ref) | 1.0 (Ref) | 1.0 (Ref) | 1.0 (Ref) | 1.0 (Ref) | 1.0 (Ref) | 1.0 (Ref) | 1.0 (Ref) | 1.0 (Ref) | 1.0 (Ref) | 1.0 (Ref) |  |
| 1 | 0.6 (0.0-26.1) | 0.6 (0.0-17.0) | 0.6 (0.0-350.6) | 1.0 (0.4-2.3) | 1.0 (0.5-2.3) | 1.0 (0.4-2.6) | 1.0 (0.5-2.1) | 1.2 (0.6-2.6) | 1.2 (0.6-2.5) | 1.6 (0.6-4.1) | 1.5 (0.5-5.3) | 1.5 (0.5-4.6) |  |
| ≥2 | 2.2 (0.8-6.2) | 0.9 (0.1-7.1) | 0.9 (0.1-10.3) | 0.7 (0.3-1.7) | 0.7 (0.3-1.8) | 0.7 (0.3-1.8) | 0.8 (0.4-1.6) | 0.8 (0.3-1.9) | 0.8 (0.4-1.6) | 0.9 (0.3-3.2) | 0.8 (0.2-3.2) | 0.8 (0.2-3.1) |  |
| *p-value* | *0.227* | *0.955* | *0.987* | *0.706* | *0.797* | *0.803* | *0.790* | *0.744* | *0.680* | *0.629* | *0.708* | *0.555* | *0.999* |
| **MOSPF-10 physical functioning scale** |  |  |  |  |  |  |  |  |  |  |  |  |  |
| 90-100 (high function) | 1.0 (Ref) | 1.0 (Ref) | 1.0 (Ref) | 1.0 (Ref) | 1.0 (Ref) | 1.0 (Ref) | 1.0 (Ref) | 1.0 (Ref) | 1.0 (Ref) | 1.0 (Ref) | 1.0 (Ref) | 1.0 (Ref) |  |
| 60-89 | 0.7 (0.1-3.2) | 0.3 (0.0-1.7) | 0.3 (0.0-2.3) | 1.5 (1.0-2.2) | 1.1 (0.7-1.7) | 1.1 (0.7-1.7) | 0.4 (0.3-0.7) | 0.4 (0.3-0.7) | 0.4 (0.3-0.7) | 2.5 (1.1-5.5) | 1.6 (0.7-3.6) | 1.6 (0.7-3.9) |  |
| 0-59 (low function) | 3.1 (0.5-18.0) | 1.9 (0.4-9.7) | 1.9 (0.4-10.4) | 1.7 (1.0-2.8) | 1.3 (0.8-2.1) | 1.3 (0.7-2.1) | 0.5 (0.3-0.8) | 0.5 (0.3-0.8) | 0.5 (0.3-0.8) | 0.8 (0.3-2.0) | 0.3 (0.1-0.9) | 0.3 (0.1-1.0) |  |
| Unspecified | 2.2 (0.2-24.9) | 0.5 (0.1-3.4) | 0.5 (0.1-3.7) | 1.2 (0.6-2.1) | 0.8 (0.5-1.5) | 0.8 (0.5-1.5) | 0.6 (0.4-1.0) | 0.5 (0.3-0.9) | 0.5 (0.3-0.9) | 2.1 (0.8-6.0) | 1.0 (0.3-3.0) | 1.0 (0.3-2.9) |  |
| *p-value* | *0.412* | *0.310* | *0.557* | *0.136* | *0.623* | *0.614* | *<0.001*** | *<0.001*** | *<0.001*** | *0.046** | *0.047** | *0.084* | *0.022** |
| **Emergency visit <31 days pre-diagnosis** |  |  |  |  |  |  |  |  |  |  |  |  |  |
| Yes | 4.0 (1.3-12.5) | 4.2 (0.9-19.9) | 4.2 (0.5-35.8) | 0.8 (0.4-1.6) | 0.7 (0.4-1.3) | 0.7 (0.3-1.4) | 0.5 (0.2-0.9) | 0.5 (0.2-0.9) | 0.5 (0.2-1.0) | 2.7 (1.3-5.6) | 2.6 (1.2-5.7) | 2.6 (1.2-5.8) |  |
| No | 1.0 (Ref) | 1.0 (Ref) | 1.0 (Ref) | 1.0 (Ref) | 1.0 (Ref) | 1.0 (Ref) | 1.0 (Ref) | 1.0 (Ref) | 1.0 (Ref) | 1.0 (Ref) | 1.0 (Ref) | 1.0 (Ref) |  |
| *p-value* | *0.017** | *0.070* | *0.188* | *0.452* | *0.238* | *0.309* | *0.028** | *0.015** | *0.041** | *0.009** | *0.018** | *0.021** | *0.005** |
| **Smoking status** |  |  |  |  |  |  |  |  |  |  |  |  |  |
| Never smoker | 1.0 (Ref) | 1.0 (Ref) | 1.0 (Ref) | 1.0 (Ref) | 1.0 (Ref) | 1.0 (Ref) | 1.0 (Ref) | 1.0 (Ref) | 1.0 (Ref) | 1.0 (Ref) | 1.0 (Ref) | 1.0 (Ref) |  |
| Former smoker (quit > 15 years) | 0.4 (0.1-0.9) | 0.2 (0.0-1.0) | 0.2 (0.0-0.8) | 1.0 (0.7-1.5) | 0.9 (0.6-1.4) | 0.9 (0.6-1.4) | 1.0 (0.7-1.3) | 0.9 (0.7-1.3) | 0.9 (0.7-1.3) | 1.5 (0.7-3.1) | 1.0 (0.5-2.1) | 1.0 (0.5-2.1) |  |
| Current/Former smoker (quit ≤ 15 years) | 1.3 (0.4-4.0) | 0.5 (0.1-2.6) | 0.5 (0.1-2.3) | 0.9 (0.6-1.4) | 0.9 (0.6-1.4) | 0.9 (0.6-1.4) | 0.9 (0.6-1.3) | 0.9 (0.6-1.3) | 0.9 (0.6-1.3) | 1.3 (0.6-2.8) | 0.8 (0.3-1.8) | 0.8 (0.3-1.8) |  |
| *p-value* | *0.089* | *0.139* | *0.088* | *0.940* | *0.922* | *0.919* | *0.793* | *0.798* | *0.786* | *0.508* | *0.757* | *0.756* | *0.583* |
| **Body Mass Index (kg/m^2^)** |  |  |  |  |  |  |  |  |  |  |  |  |  |
| Underweight/Normal (<25) | 1.0 (Ref) | 1.0 (Ref) | 1.0 (Ref) | 1.0 (Ref) | 1.0 (Ref) | 1.0 (Ref) | 1.0 (Ref) | 1.0 (Ref) | 1.0 (Ref) | 1.0 (Ref) | 1.0 (Ref) | 1.0 (Ref) |  |
| Overweight/Obese (≥25) | 0.6 (0.2-1.5) | 0.7 (0.3-2.1) | 0.7 (0.3-1.5) | 1.1 (0.8-1.6) | 1.1 (0.8-1.6) | 1.1 (0.8-1.6) | 1.1 (0.8-1.5) | 1.1 (0.8-1.5) | 1.1 (0.8-1.4) | 0.6 (0.3-1.2) | 0.6 (0.3-1.3) | 0.6 (0.3-1.4) |  |
| Unspecified | 0.3 (0.0-8.3) | 0.4 (0.0-11.1) | 0.4 (0.0-105.3) | 1.2 (0.6-2.2) | 1.5 (0.8-2.7) | 1.5 (0.8-2.6) | 0.8 (0.4-1.6) | 0.9 (0.5-1.8) | 0.9 (0.5-1.9) | 2.1 (0.8-5.4) | 2.0 (0.7-6.2) | 2.0 (0.7-5.8) |  |
| *p-value* | *0.458* | *0.775* | *0.663* | *0.838* | *0.483* | *0.417* | *0.450* | *0.848* | *0.843* | *0.022** | *0.093* | *0.062* | *0.347* |
| **Ever had FOBT** |  |  |  |  |  |  |  |  |  |  |  |  |  |
| Yes | 1.0 (Ref) | 1.0 (Ref) | 1.0 (Ref) | 1.0 (Ref) | 1.0 (Ref) | 1.0 (Ref) | 1.0 (Ref) | 1.0 (Ref) | 1.0 (Ref) | 1.0 (Ref) | 1.0 (Ref) | 1.0 (Ref) |  |
| No | 4.0 (0.7-22.6) | 23.9 (2.4-242.4) | 23.9 (0.7-772.3) | 0.8 (0.6-1.2) | 0.9 (0.6-1.4) | 0.9 (0.6-1.4) | 1.0 (0.7-1.5) | 1.0 (0.7-1.4) | 1.0 (0.7-1.4) | 2.5 (0.7-9.3) | 3.6 (1.0-12.5) | 3.6 (0.9-13.9) |  |
| *p-value* | *0.115* | *0.007** | *0.073* | *0.329* | *0.702* | *0.711* | *0.865* | *0.845* | *0.828* | *0.168* | *0.044** | *0.064* | *0.067* |
| **Ever had sigmoidoscopy/colonoscopy** | |  |  |  |  |  |  |  |  |  |  |  |  |
| Yes | 1.0 (Ref) | 1.0 (Ref) | 1.0 (Ref) | 1.0 (Ref) | 1.0 (Ref) | 1.0 (Ref) | 1.0 (Ref) | 1.0 (Ref) | 1.0 (Ref) | 1.0 (Ref) | 1.0 (Ref) | 1.0 (Ref) |  |
| No | 1.1 (0.1-10.7) | 0.9 (0.1-7.8) | 0.9 (0.2-5.4) | 1.0 (0.7-1.5) | 1.1 (0.7-1.7) | 1.1 (0.7-1.7) | 1.3 (0.9-2.0) | 1.4 (0.9-2.1) | 1.4 (0.9-2.1) | 0.5 (0.2-1.3) | 0.5 (0.2-1.3) | 0.5 (0.2-1.3) |  |
| *p-value* | *0.960* | *0.956* | *0.947* | *0.992* | *0.690* | *0.685* | *0.213* | *0.168* | *0.171* | *0.186* | *0.139* | *0.146* | *0.320* |
| **Place of residence** |  |  |  |  |  |  |  |  |  |  |  |  |  |
| Major City | 1.0 (Ref) | 1.0 (Ref) | 1.0 (Ref) | 1.0 (Ref) | 1.0 (Ref) | 1.0 (Ref) | 1.0 (Ref) | 1.0 (Ref) | 1.0 (Ref) | 1.0 (Ref) | 1.0 (Ref) | 1.0 (Ref) |  |
| Other | 2.4 (0.7-8.5) | 6.9 (1.4-34.2) | 6.9 (0.9-53.5) | 0.8 (0.5-1.2) | 0.8 (0.5-1.2) | 0.8 (0.5-1.2) | 0.8 (0.6-1.1) | 0.8 (0.6-1.1) | 0.8 (0.6-1.1) | 1.1 (0.6-2.1) | 1.3 (0.7-2.8) | 1.3 (0.7-2.6) |  |
| *p-value* | *0.169* | *0.018** | *0.064* | *0.323* | *0.307* | *0.299* | *0.134* | *0.147* | *0.121* | *0.806* | *0.420* | *0.373* | *0.087* |
| **Area-level SES** |  |  |  |  |  |  |  |  |  |  |  |  |  |
| Quintile 4 or 5 (least disadvantaged) | 1.0 (Ref) | 1.0 (Ref) | 1.0 (Ref) | 1.0 (Ref) | 1.0 (Ref) | 1.0 (Ref) | 1.0 (Ref) | 1.0 (Ref) | 1.0 (Ref) | 1.0 (Ref) | 1.0 (Ref) | 1.0 (Ref) |  |
| Quintile 2 or 3 | 5.3 (1.2-23.9) | 25.3 (3.4-190.6) | 25.3 (2.0-323.2) | 1.5 (1.0-2.3) | 1.5 (1.0-2.3) | 1.5 (1.0-2.3) | 0.9 (0.6-1.3) | 1.0 (0.7-1.3) | 1.0 (0.7-1.3) | 1.3 (0.6-2.8) | 1.3 (0.6-3.0) | 1.3 (0.6-2.8) |  |
| Quintile 1 (most disadvantaged) | 3.9 (0.7-22.8) | 3.1 (0.5-20.8) | 3.1 (0.4-25.5) | 1.6 (1.0-2.9) | 1.5 (0.9-2.5) | 1.5 (0.9-2.5) | 0.7 (0.4-1.1) | 0.6 (0.4-1.1) | 0.6 (0.4-1.0) | 2.0 (0.8-5.2) | 1.2 (0.5-3.2) | 1.2 (0.4-3.3) |  |
| *p-value* | *0.099* | *0.004** | *0.042** | *0.162* | *0.184* | *0.196* | *0.206* | *0.152* | *0.140* | *0.331* | *0.832* | *0.797* | *0.095* |
| **Highest qualification** |  |  |  |  |  |  |  |  |  |  |  |  |  |
| University degree or higher | 1.0 (Ref) | 1.0 (Ref) | 1.0 (Ref) | 1.0 (Ref) | 1.0 (Ref) | 1.0 (Ref) | 1.0 (Ref) | 1.0 (Ref) | 1.0 (Ref) | 1.0 (Ref) | 1.0 (Ref) | 1.0 (Ref) |  |
| School/Higher school/Trade/Certificate | 0.6 (0.1-2.5) | 0.7 (0.1-3.8) | 0.7 (0.1-5.9) | 1.1 (0.7-1.8) | 1.0 (0.6-1.7) | 1.0 (0.6-1.7) | 0.8 (0.6-1.2) | 0.8 (0.5-1.1) | 0.8 (0.6-1.1) | 1.8 (0.7-4.3) | 1.2 (0.4-3.2) | 1.2 (0.4-3.1) |  |
| No school certificate or qualification | 0.2 (0.0-1.6) | 0.3 (0.0-2.7) | 0.3 (0.0-5.4) | 1.5 (0.8-2.7) | 1.3 (0.7-2.5) | 1.3 (0.7-2.5) | 0.7 (0.4-1.3) | 0.8 (0.5-1.3) | 0.8 (0.5-1.4) | 0.8 (0.2-2.9) | 0.7 (0.2-2.7) | 0.7 (0.2-2.7) |  |
| *p-value* | *0.317* | *0.500* | *0.673* | *0.278* | *0.462* | *0.430* | *0.481* | *0.321* | *0.289* | *0.153* | *0.561* | *0.551* | *0.565* |
| **Private health insurance** |  |  |  |  |  |  |  |  |  |  |  |  |  |
| Yes | 1.0 (Ref) | 1.0 (Ref) | 1.0 (Ref) | 1.0 (Ref) | 1.0 (Ref) | 1.0 (Ref) | 1.0 (Ref) | 1.0 (Ref) | 1.0 (Ref) | 1.0 (Ref) | 1.0 (Ref) | 1.0 (Ref) |  |
| No | 0.8 (0.3-2.2) | 0.8 (0.2-2.7) | 0.8 (0.2-3.3) | 0.8 (0.6-1.2) | 0.7 (0.5-1.0) | 0.7 (0.5-1.0) | 0.7 (0.5-0.9) | 0.6 (0.5-0.9) | 0.6 (0.5-0.8) | 2.1 (1.1-3.9) | 1.6 (0.8-3.2) | 1.6 (0.8-3.3) |  |
| *p-value* | *0.733* | *0.749* | *0.784* | *0.310* | *0.046** | *0.040** | *0.014** | *0.003*** | *0.002*** | *0.028** | *0.207* | *0.215* | *0.166* |
| **Married or de-facto** |  |  |  |  |  |  |  |  |  |  |  |  |  |
| Yes | 1.0 (Ref) | 1.0 (Ref) | 1.0 (Ref) | 1.0 (Ref) | 1.0 (Ref) | 1.0 (Ref) | 1.0 (Ref) | 1.0 (Ref) | 1.0 (Ref) | 1.0 (Ref) | 1.0 (Ref) | 1.0 (Ref) |  |
| No | 0.7 (0.2-2.7) | 1.0 (0.3-3.6) | 1.0 (0.2-4.7) | 0.8 (0.5-1.2) | 0.8 (0.6-1.2) | 0.8 (0.6-1.3) | 1.1 (0.8-1.5) | 1.1 (0.8-1.5) | 1.1 (0.8-1.5) | 1.2 (0.7-2.1) | 1.3 (0.7-2.6) | 1.3 (0.7-2.4) |  |
| *p-value* | *0.576* | *0.960* | *0.967* | *0.257* | *0.393* | *0.406* | *0.682* | *0.476* | *0.450* | *0.473* | *0.432* | *0.393* | *0.638* |
| **Language other than English** |  |  |  |  |  |  |  |  |  |  |  |  |  |
| Yes | 0.6 (0.0-11.1) | 0.9 (0.1-8.4) | 0.9 (0.0-40.6) | 1.3 (0.7-2.4) | 1.1 (0.6-2.0) | 1.1 (0.6-2.0) | 0.5 (0.3-0.9) | 0.5 (0.3-1.0) | 0.5 (0.3-0.9) | 0.8 (0.3-2.2) | 1.1 (0.4-3.4) | 1.1 (0.4-3.0) |  |
| No | 1.0 (Ref) | 1.0 (Ref) | 1.0 (Ref) | 1.0 (Ref) | 1.0 (Ref) | 1.0 (Ref) | 1.0 (Ref) | 1.0 (Ref) | 1.0 (Ref) | 1.0 (Ref) | 1.0 (Ref) | 1.0 (Ref) |  |
| *p-value* | *0.722* | *0.914* | *0.950* | *0.405* | *0.696* | *0.714* | *0.014** | *0.037** | *0.027** | *0.714* | *0.835* | *0.813* | *0.318* |
