# Supplementary material for: Colon and rectal cancer treatment patterns and their associations with clinical, sociodemographic and lifestyle characteristics: analysis of the Australian 45 and Up Study cohort
Source: BMC Cancer. 2023 Jan 18;23:60. doi: 10.1186/s12885-023-10528-8 (PMC9845101; doi:10.1186/s12885-023-10528-8)
Supplement: Supplementary file 14 — Additional file 14. Details on an emergency presentation prior to cancer diagnosis. [file 12885_2023_10528_MOESM14_ESM.docx]

**Additional file 14. Details on an emergency presentation prior to cancer diagnosis**

Our study found 22.8% and 10.4% of colon and rectal cancer cases, respectively, visited an emergency department in the month before their cancer diagnosis. Emergency department visits were associated with a higher hazard of no treatment and death within 2 years after diagnosis. However, using the available data for this cohort, we cannot determine whether the emergency department visits are related to the cancer diagnosis. In particular, there may be interdependencies between emergency department visits and other characteristics, such as presence of specific comorbidities; investigation of such complex relationships was beyond the scope of the current study.

Previous Australian and international studies have found that an emergency department visit prior to a cancer diagnosis may be associated with poorer outcomes [1-4]. One Scottish study indicated colorectal cancer cases presenting via an emergency route were older and less likely to be treated with curative intent, and had lower survival than cases presenting via other routes [2, 5]. There are few Australian studies examining characteristics associated with an emergency presentation prior to cancer diagnosis, especially distinguishing emergency presentations that are related to cancer symptoms or part of the pathway to diagnosis. Future research investigating these factors may provide insights to improve diagnostic pathways.

**References**

1. McPhail S, Swann R, Johnson SA, Barclay ME, Abd Elkader H, Alvi R, Barisic A, Bucher O, Clark GRC, Creighton N *et al*: **Risk factors and prognostic implications of diagnosis of cancer within 30 days after an emergency hospital admission (emergency presentation): an International Cancer Benchmarking Partnership (ICBP) population-based study**. *The Lancet Oncology* 2022.
2. Zhou Y, Abel GA, Hamilton W, Pritchard-Jones K, Gross CP, Walter FM, Renzi C, Johnson S, McPhail S, Elliss-Brookes L *et al*: **Diagnosis of cancer as an emergency: a critical review of current evidence**. *Nat Rev Clin Oncol* 2017, **14**(1):45-56
3. Yap S, Goldsbury D, Yap ML, Yuill S, Rankin N, Weber M, Canfell K, O’Connell DL: **Patterns of care and emergency presentations for people with non-small cell lung cancer in New South Wales, Australia: A population-based study**. *Lung Cancer* 2018, **122**:171-179.
4. Ngo P, Goldsbury DE, Karikios D, Yap S, Yap ML, Egger S, O'Connell DL, Ball D, Fong KM, Pavlakis N *et al*: **Lung cancer treatment patterns and factors relating to systemic therapy use in Australia**. *Asia-Pacific Journal of Clinical Oncology* 2021.
5. McArdle CS, Hole DJ: **Emergency presentation of colorectal cancer is associated with poor 5-year survival**. *British Journal of Surgery* 2004, **91**(5):605-609.
